# Differential Expression of Estrogen-Responsive Genes in Women with Psoriasis

**DOI:** 10.3390/jpm11090925

**Published:** 2021-09-17

**Authors:** Vladimir Sobolev, Anna Soboleva, Elena Denisova, Malika Denieva, Eugenia Dvoryankova, Elkhan Suleymanov, Olga V. Zhukova, Nikolay Potekaev, Irina Korsunskaya, Alexandre Mezentsev

**Affiliations:** 1 Centre of Theoretical Problems of Physico-Chemical Pharmacology, Russian Academy of Sciences, Russian Academy of Sciences, 119334 Moscow, Russia; annasobo@mail.ru (A.S.); evdenissova@rambler.ru (E.D.); edvoriankova@gmail.com (E.D.); marykor@bk.ru (I.K.); mesentsev@yahoo.com (A.M.); 2 Scientific Research Institute of Human Morphology, 3 Tsurupa Street, 117418 Moscow, Russia; 3 Moscow Scientific and Practical Center of Dermatovenereology and Cosmetology, 119071 Moscow, Russia; klinderma@inbox.ru (O.V.Z.); klinderma@mail.ru (N.P.); 4 Department of Polyclinic Therapy, Chechen State University, 366007 Grozny, Russia; denieva54@mail.ru; 5 P. Hertsen Moscow Oncology Research Institute, National Medical Research Radiological Centre of the Ministry of Health of the Russian Federation, 3, 2 Botkinskiy Proezd, 125284 Moscow, Russia; Docseasur@mail.ru

**Keywords:** psoriasis, proteome analysis, estrogen, menopause

## Abstract

In women, the flow of psoriasis is influenced by each phase of a woman’s life cycle. According to previous findings, significant changes in the levels of sex hormones affect the severity of the disease. **Aim**: The aim of this study was to identify the estrogen-responsive genes that could be responsible for the exacerbation of psoriasis in menopausal women. **Methods**: Skin samples of lesional skin donated by psoriasis patients (n = 5) were compared with skin samples of healthy volunteers (n = 5) using liquid chromatography–tandem mass spectrometry (LC–MS/MS). The set of differentially expressed proteins was subjected to protein ontology analysis to identify differentially expressed estrogen-responsive proteins. The expression of discovered proteins was validated by qPCR and ELISA on four groups of female participants. The first group included ten psoriasis patients without menopause; the second included eleven postmenopausal patients; the third included five healthy volunteers without menopause; and the fourth included six postmenopausal volunteers. Moreover, the participants’ blood samples were used to assess the levels of estradiol, progesterone, and testosterone. **Results**: We found that the levels of estradiol and progesterone were significantly lower and the levels of testosterone were significantly higher in the blood of patients compared to the control. The protein ontology analysis of LC–MS/MS data identified six proteins, namely HMOX1, KRT19, LDHA, HSPD1, MAPK1, and CA2, differentially expressed in the lesional skin of female patients compared to male patients. ELISA and qPCR experiments confirmed differential expression of the named proteins and their mRNA. The genes encoding the named proteins were differentially expressed in patients compared to volunteers. However, *KRT19* and *LDHA* were not differentially expressed when we compared patients with and without menopause. All genes, except *MAPK1*, were differentially expressed in patients with menopause compared to the volunteers with menopause. *HMOX1*, *KRT19*, *HSPD1*, and *LDHA* were differentially expressed in patients without menopause compared to the volunteers without menopause. However, no significant changes were found when we compared healthy volunteers with and without menopause. **Conclusion**: Our experiments discovered a differential expression of six estrogen-controlled genes in the skin of female patients. Identification of these genes and assessment of the changes in their expression provide insight into the biological effects of estrogen in lesional skin. The results of proteomic analysis are available via ProteomeXchange with identifier PXD021673.

## 1. Introduction

Psoriasis is an immune-mediated disease that is driven by T_h1_ and T_h17_ cells [1]. The incidence of psoriasis is similar in men and women. The mean age at onset of psoriasis presentation ranges between 15 and 20 years of age and the second peak occurs at the ages of 55–60 [2]. It is well-documented that the endogenous factors such as hormonal changes may trigger psoriasis [3]. In women, the severity of psoriasis is influenced by each phase of a woman’s life cycle and the disease frequency tends to peak during puberty, postpartum, and menopause. In contrast, the patients’ condition often improves during pregnancy [4].

Although puberty is the period of life when the first signs of psoriasis often appear, there is a lack of evidence that female sex hormones trigger the disease. In fact, an increased production of estradiol (E2) and progesterone (PG) during the menstrual cycle has anti-inflammatory effects [5]. Moreover, PG shifts the balance between Th_1_ and Th_2_ responses toward Th_2_ [6]. In pregnancy, an increased production of estriol and PG often results in an improvement of symptoms in a majority of psoriasis patients.

However, psoriasis exacerbates in the first months of the postpartum period and the body surface area covered by psoriasis (BSA) significantly increases [7]. There is also a negative correlation between the levels of estrogen (ES) and BSA [4]. Moreover, prolactin (PRL) released by the pituitary gland of the brain stimulates immunity [8]. Respectively, patients with hyperprolactinemia present with many different clinical manifestations, including psoriasis [9]. In addition, there is a correlation of the PRL level and disease severity [9].

In perimenopause, the remaining aging follicles produce less inhibin and ES [10]. Since they are suppressed, the synthesis and secretion of the follicle-stimulating hormone (FSH) and luteinizing hormone (LH) gradually increase and a higher production of FSH stabilizes the level of ES. For this reason, ES can be slightly elevated for a limited period of time. Then, ES drops because there are less ES-producing cells in the ovary and they require more FSH to produce the same amount of ES. Consequently, the level of FSH continues to increase due to an existence of the negative feedback between the synthesis of ES and production of FSH [11], and it is often accompanied by an exacerbation of psoriasis [8].

Our own observations suggest that young women diagnosed with psoriasis have lower ES levels compared to healthy controls [12,13]. In this paper, we aim to identity the genes that could be targeted by ES in the lesional and uninvolved skin of female patients.

## 2. Materials and Methods

### 2.1. Ethics Statement

All samples were obtained with informed written consent from healthy volunteers and psoriasis patients in accordance with Declaration of Helsinki principles. All protocols were approved by an institutional review board (I.I. Mechnikov Institute of Vaccines and Sera, Moscow, Russia).

### 2.2. Clinical Samples

Skin biopsies for LC–MS/MS study were obtained from 5 healthy volunteers (MS volunteers), namely 2 males and 3 females between the ages of 39 and 79 years (mean age: 61.6 years), and from an equal number of psoriasis patients (MS patients), namely 3 males and 2 females between the ages of 30 and 68 years (mean age: 49.2 years). Skin biopsies for qPCR and ELISA assays were obtained from female participants: 20 psoriasis patients and 11 healthy volunteers that we identified as qPCR/ELISA participants. To distinguish the changes in gene expression caused by the disease from ones caused by menopause, the participants were divided in four groups. The first group included 10 patients without menopause between the ages of 19 and 43 years (mean age: 31.5 years). The second group included 10 patients with menopause between the ages of 46 and 54 years (mean age: 50.2 years). The third group included 6 healthy volunteers without menopause between the ages of 26 and 38 years (mean age: 31 years). The fourth group included 5 healthy volunteers with menopause between the ages of 43 and 57 (mean age: 50.8 years). The additional details on participants of this study can be found in Table 1.

The patients that participated in our study discontinued topical treatment for 1 week prior to the biopsy collection (2 week in the case of systemic therapies). Each patient donated two 4 mm punch biopsies of lesional and uninvolved skin following a local anesthesia. Biopsies of uninvolved skin were taken at least 6 cm away from the nearest skin lesion. The collected biopsies were flash frozen in liquid nitrogen and stored at −80 °C until processing. Blood (3 mL) was withdrawn by venipuncture without anticoagulant at day 3 or 4 of the menstrual cycle. Serum was separated, divided into aliquots, and stored frozen at −20 °C until needed.

### 2.3. Preparation of Skin Samples for LC–MS/MS Experiments

Each sample was washed twice with 0.5 mL of phosphate-buffered saline. The samples were homogenized by mechanical disruption in liquid nitrogen. To prepare the protein samples, sodium deoxycholate (SDS) lysis as well as reduction and alkylation buffer pH 8.5, which contained 100 mM TRIS, 1% (*w*/*v*) SDS, 40 mM 2-chloroacetamide, and 10 mM TCEP, were added to the homogenized samples. The samples were sonicated and boiled for 10 min. Then, the protein concentration was determined by Bradford assay and the equal volumes of 1% trypsin solution (*w*/*v*) prepared in 100 mM TRIS pH 8.5 were added.

After overnight digestion at 37 °C, peptides were acidified with 1% trifluoroacetic acid (TFA). The samples (2 × 20 μg) were loaded on 14-gauge StageTips containing 2 layers of SDB-RPS discs. Respectively, 2 tips per a sample were used. The tips were consequently washed with equal volumes of ethyl acetate, 100 μL of 1% TFA prepared in ethyl acetate, and 100 μL of 0.2% TFA. After each washing, the excess of liquid was removed by centrifugation (300 g; 1.5 min.) Then, the peptides were eluted with 60 μL of 5% NH_4_OH prepared in 80% acetonitrile. The eluates were vacuum-dried and stored at −80 °C. Prior to the experiment, the vacuum-dried samples were dissolved in 2% acetonitrile/0.1% TFA buffer and sonicated for 2 min.

### 2.4. LC-MS/MS Analysis

The reverse-phase chromatography was performed on the Ultimate 3000 Nano LC System (Thermo Fisher Scientific, Waltham, MA, USA) coupled to the Q Exactive Plus benchtop Orbitrap mass spectrometer (Thermo Fisher Scientific, Waltham, USA) using a chip-based nanoelectrospray source (Thermo Fisher Scientific, Waltham, USA). Samples prepared in the loading buffer (0.1% TFA and 2% acetonitrile in water) were loaded on the Inertsil ODS3 (GLSciences, Torrance, USA) trap column (0.1 × 20 mm, 3 μm) at 10 μL/min and separated on the Reprosil PUR C18AQ (Dr. Maisch, Germany) fused-silica column (0.1 × 500 mm, 1,9 μm) with a linear gradient of 3–35% buffer B (0.1% formic acid, 80% acetonitrile in water) for 55 min; 35–55% B for 5 min; and 55–100% B for 1 min at a flow rate of 440 nL/min. Prior to injection of the next sample, the column was washed with buffer B for 5 min and re-equilibrated with buffer A (0.1% formic acid and 3% acetonitrile in water) for 5 min.

Peptides were analyzed on the mass spectrometer with one full scan (350–2000 *m*/*z*, *R* = 70,000 at 200 *m*/*z*) at a target of 3 × 10^6^ ions and a maximum ion fill-time of 50 ms, followed by up to 10 data-dependent MS/MS scans with higher-energy collisional dissociation (HCD) (target 1 × 10^5^ ions, max ion fill time 45 ms, isolation window 1.4 *m*/*z*, normalized collision energy (NCE) 27%) detected in the Orbitrap (*R* = 17,500 at fixed first mass 100 *m*/*z*). Other settings included: charge exclusion: unassigned, 1, and more than 6; peptide match–preferred; excluded isotopes–on; and the dynamic exclusion of 40 s was enabled.

### 2.5. Analysis of LC–MS/MS Data

Label-free protein quantification was performed using MaxQuant software version 1.5.6.5 (Max Plank Institute of Biochemistry, Planegg, Germany) and a common contaminants database by the Andromeda search engine [14] with cysteine carbamidomethylation as a fixed modification was used. Oxidation of methionine and protein N-terminal acetylation were used as variable modifications. Peak lists were searched against the human protein sequences extracted from the Uniprot (28.06.19) database. The false discovery rate (FDR) was set to 0.01 for both proteins and peptides with a minimum length of seven amino acids. Peptide identification was performed with an allowed initial precursor mass deviation of up to 20 ppm and an allowed fragment mass deviation of 20 ppm. Downstream bioinformatics analysis was performed using Perseus software, version 1.5.5.1 (Max Plank Institute of Biochemistry, Planegg, Germany). Protein groups only identified by site, only from peptides identified also in the reverse database, or those belonging to the common contaminants database were excluded from the analyses. For Student’s *t*-test, missing values were imputed with a width of 0.3 and a downshift of 1.8 over the total matrix. Two sample tests were performed in Perseus with s0 set to 0. Label-free quantification was performed using a minimum ratio count of 1. The protein levels were assessed by the iBAQ (intensity-based absolute quantification) method using MaxQuant software. To determine the relative abundance of identified proteins in the samples (riBAQ), we divided the obtained iBAQ values by the sum of all iBAQ values and expressed this ratio as percentage. The results were analyzed using Venn diagrams. Protein ontology analysis of the differentially expressed proteins (PO) was performed on gene ontology terms to catalog the biological processes using DAVID Bioinformatics resources, 6.7 (Frederick National Laboratory for Cancer Research, Frederick, MD, USA). The mass spectrometry proteomics data were deposited to the ProteomeXchange Consortium via the PRIDE [15] partner repository with the dataset identifier PXD021673.

### 2.6. Quantitative PCR

The gene expression analysis of *HMOX1*, *KRT19*, *LDHA*, *HSPD1*, *MAPK1*, and *CA2* in lesional and healthy skin was performed using the method of quantitative PCR (qPCR). The Qiagen RNeasy Mini Kit with spin columns was used to isolate the total RNA from the skin. The isolated total RNA was treated with DNase (Qiagen, Hilden, Germany) to remove the traces of genomic DNA. RNA concentration was measured with NanoDrop 1000 (Thermo Fisher Scientific, Waltham, USA). The M-MLV kit (Promega, Madison, WI, USA) was used for the reverse transcription with oligo-dT (DNA-Synthes, Moscow, Russia) primers according to the manufacturer’s protocol.

The primers used in the qPCR experiments (Table 2) were designed in Primer blast (NCBI, USA), checked with the Multiple primer analyzer (Thermo Fisher Scientific, Waltham, USA) for the formation of potential secondary structures and dimers, and synthesized by DNA-Synthes (Moscow, Russia). The experiments were performed in the CFX96 Touch real-time DNA detection system (Bio-Rad, Hercules, CA, USA) using the SYBR-Green master mix supplied by Evrogen (Moscow, Russia) according to the manufacturer’s instructions. The following conditions were used to amplify the DNA: 4 min at 95 °C, followed by 40 cycles of consequent incubations at 94 °C for 15 s and 60 °C for 30 s. Each reaction was run in triplicates. 18S RNA was used as a housekeeping gene to normalize the expression levels of the target genes.

The results were analyzed using the standard 2^−ΔΔCT^ method [16] to compare the levels of expressed genes. Each ΔCt value was calculated as ΔCt = Ct (tested gene) − Ct (housekeeping gene). ΔΔCt was calculated as ΔΔCt = ΔCt (sample of psoriatic patient) − ΔCt (sample of healthy individual). The experiments were repeated three times for each sample.

### 2.7. ELISA

The protein expression of KRT19 and HSPD1 in lesional and healthy skin was evaluated using ELISA kits (MyBiosource, Inc., San Diego, CA, USA, MBS2703060 and MBS450548, respectively) according to the manufacturer’s protocol. Briefly, tissue samples were prepared in lysis buffer (25 mg per 1 mL), homogenized, centrifuged (10,000 g; 5 min; 4 °C), aliquoted, and stored at −80 °C. Before the experiment, samples, blanks, and standards were loaded on 96-well plates and incubated for 1 h at 37 °C. Then, the solutions were replaced by detection reagent A and incubation continued for the same period of time. After washing with wash solution (3 × 2 min) detection reagent B was added and incubation continued for another 20 min. Then, the wells were washed again (5 × 2 min). The presence of antigen was visualized with the chromogenic substrate 3,3′,5,5′-tetramethylbenzidine (TMB) and assayed using a microplate reader (Bio-Rad, Hercules, CA, USA) at the wavelength 450 nm. The antigen was quantified with a standard curve generated with standards of known concentrations.

The blood levels of E2, PG, and TS were analyzed using ELISA kits (Diagnostics Biochem Canada, Inc., London, ON, Canada CAN-F-430, CAN-PRE-4500, and CAN-TE-250) according to the instructions provided by the manufacturer. To perform an assay, the calibrator, control, and specimen samples were loaded on 96-well plates and mixed with aliquots of horse radish peroxidase (HRP) conjugated to a tested hormone. The plate was incubated for 1 h at room temperature on a shaker (200 rpm). After washing 3 times with provided washing buffer, TMB was added and the incubation continued for another 10–15 min. Then, the presence of a tested hormone was revealed by measuring the absorbance using a microplate reader (Bio-Rad, Hercules, USA) at the wavelength 450 nm. The antigen was quantified with a standard curve generated with standards of known concentrations.

### 2.8. Statistical Analysis

Due to small sample size, the data of LC–MS/MS were analyzed with non-parametric statistics using the Mann–Whitney U test. The Mann–Whitney U test was also used to evaluate gender-related differences in protein expression. Data variability was analyzed in R using the “prcomp” function. Two-sided unpaired Student’s *t*-test was used to analyze the data of the qPCR and ELISA studies that were performed on larger groups of participants. In all cases, differences were considered statistically significant when *p* < 0.05.

## 3. Results

### 3.1. The Blood of Female Psoriasis Patients Contains Less Estradiol and Progesterone, and More Testosterone Compared to the Healthy Volunteers

**Figure 1:**
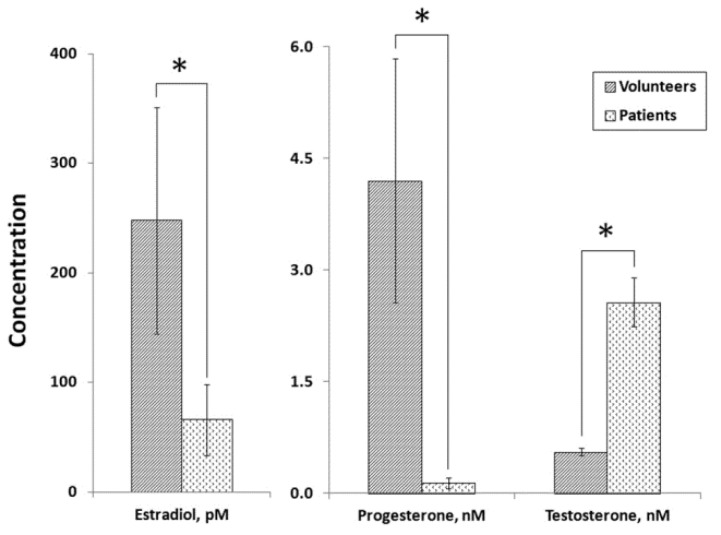
The levels of sex hormones in the blood of psoriasis patients and healthy volunteers that participated in the qPCR and ELISA experiments. The following individuals participated in these experiments: psoriasis patients without menopause (n = 10); psoriasis patients with menopause (n = 10); healthy volunteers without menopause (n = 6); and healthy volunteers with menopause (n = 5). * *p* < 0.05 when patients compared to healthy volunteers.

The analysis of blood samples (Figure 1) revealed significant differences in the levels of sex hormones between qPCR/ELISA psoriasis patients (n = 20) and qPCR/ELISA healthy volunteers (n = 11). The levels of E2 and PG were significantly higher in healthy volunteers (*p* = 0.042 and 0.001, respectively). In contrast, the level of TS was significantly higher in patients (*p* = 2 × 10^−4^). In turn, the blood of participants with menopause, being either healthy volunteers or patients, contained less E2 and PG but more TS compared to their normally menstruating counterparts (Table 3).

### 3.2. LC–MS/MS Study Identifies Six Estrogen-Responsive Proteins That Are Differentially Expressed in Male and Female Psoriatic Skin

**Figure 2:**
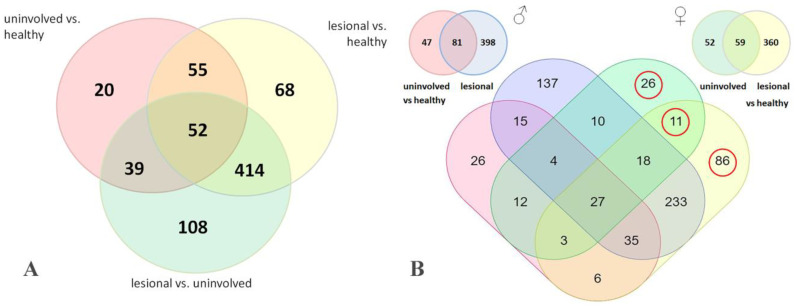
Venn diagram comparing DEPs in the donated skin samples as assessed by LC/MS–MS analysis. (**A**) Paired comparison of the samples obtained from lesional and uninvolved skin of the same psoriasis patients (n = 5) and skin of healthy volunteers (n = 5). (**B**) Analysis of gender-specific changes in protein expression. Samples of male (n = 3) and female (n = 2) psoriatic skin were compared to the skin of healthy volunteers (n = 5). The numbers indicated in the diagram are the numbers of DEPs in the compared groups of samples (*p* < 0.05). DEPs chosen for PO analysis are encircled. The data were compared using the Mann–Whitney U test.

To identify a possible gender-specific response to the disease, we analyzed skin samples donated by MS psoriasis patients (n = 5) and MS healthy volunteers (n = 5) of both genders using LC/MS–MS. The analysis demonstrated that 756 proteins were differentially expressed in patients’ lesional and uninvolved skin. The distribution of DEPs between the groups of samples is shown on a Venn diagram (Figure 2A). Samples of male lesional and uninvolved skin (n = 3) contained 479 and 128 DEPs (Figure 2B) compared to the skin of healthy volunteers (n = 5). Samples of female lesional and uninvolved skin (n = 2) contained 419 and 111 DEPs (Figure 2B) compared to the skin of healthy volunteers. Their paired comparison revealed 123 proteins that were differentially expressed in female skin and were not present in male skin (Supplementary Table S1). Particularly, 26 and 86 proteins were differentially expressed in female uninvolved and lesional skin. Moreover, 11 proteins were differentially expressed in both groups of samples (Figure 2B).

The following protein ontology analysis of 123 proteins differentially expressed in female lesional and uninvolved skin performed on GO terms revealed 14 overrepresented biological processes (Table 4) including GO:0043627, which is the response to estrogen enriched by six DEPs, namely HMOX1, KRT19, LDHA, HSPD1, MAPK1, and CA2 (*p* = 0.005; FDR = 0.005). Four identified proteins, namely HMOX1, KRT19, LDHA, and MAPK1, were differentially expressed in female lesional skin, whereas HSPD1 and CA2 were also differentially expressed in female uninvolved skin. Among the mentioned six DEPs, CA2 was the only less abundant protein in patients’ samples, whereas the others were more abundant in patients’ skin samples compared to healthy skin. A similar analysis of the proteins differentially expressed in male lesional and uninvolved skin and were not differentially expressed in lesional and uninvolved female skin (Supplementary Table S1) revealed 11 overrepresented biological processes (Table 5).

### 3.3. The Identified Estrogen-Responsive Genes Were Differentially Expressed in Menopausal and Non-Menopausal Patients, and Their Expression Was Influenced by the Disease

**Figure 3:**
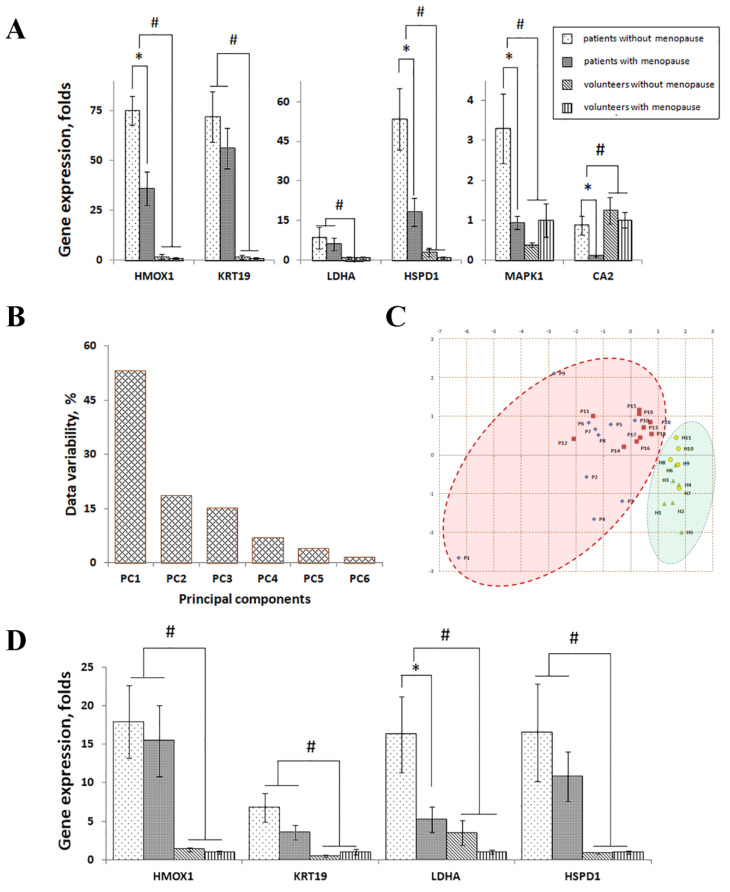
The expression of identified estrogen-responsive genes in clinical samples of psoriasis patients and healthy volunteers assessed by qPCR. (**A**). The levels of gene expression in the samples of patients’ lesional skin and in the skin of healthy volunteers. (**B**). Assessment of a variation in gene expression in the lesional skin of psoriasis patients and healthy volunteers by principle component analysis. (**C**). The plot of two first principal components (PC1 and PC2). Different groups are indicated by data points of different colors and shapes: group 1 is represented by blue diamonds; group 2 is represented by red squares; group 3 is represented by green triangles; and group 4 is represented by yellow circles. (**D**). The expression of identified estrogen-responsive genes in the PBMC obtained from the blood of patients and healthy volunteers. The following individuals participated in these experiments: psoriasis patients without menopause (n = 10); psoriasis patients with menopause (n = 10); healthy volunteers without menopause (n = 6); and healthy volunteers with menopause (n = 5). * *p* < 0.05 when women without menopause compared to women with menopause. ^#^ *p* < 0.05 when patients compared to healthy volunteers. Gene expression in menopausal patients was set equal to 1.

Due to the small sample size of the performed LC–MS/MS study, we confirmed the differential expression of the identified estrogen-responsive proteins (ERPs) in women using qPCR and ELISA. The analysis of gene expression in skin samples by qPCR revealed that the genes encoding the identified ERPs were differentially expressed in lesional skin of qPCR/ELISA patients (n = 20) compared to the skin of qPCR/ELISA healthy volunteers (n = 11). Five identified genes, namely *HMOX1* (38.97 ± 4.91; *p* = 1.30 × 10^−^^7^), *KRT19* (45.90 ± 5.86; *p* = 1.52 × 10^−^^7^), *LDHA* (7.30 ± 2.55; *p* = 0.01), *HSPD1* (17.32 ± 3.57; *p* = 1.07 × 10^−^^4^), and *MAPK1* (3.20 ± 0.77; *p* = 0.01), were induced, whereas *CA2* (0.43 ± 0.13; *p* = 0.01) was suppressed in lesional skin (Figure 3).

The expression profiles in qPCR/ELISA patients with and without menopause (Figure 3) were the same, i.e., the genes upregulated in patients without menopause (n = 10) were also upregulated in patients with menopause (n = 10) and vice versa, compared to qPCR/ELISA healthy volunteers (n = 11). All six identified ERGs had higher expression in patients without menopause compared to patients with menopause. Moreover, when patients with and without menopause were compared to each other, the changes in the expression of four genes, namely *HMOX1* (*p* = 0.001), *HSPD1* (*p* = 0.008), *CA2* (*p* = 0.006), and *MAPK1* (*p* = 0.012), were significant. In contrast, we did not see significant changes in gene expression (Figure 3) when we compared qPCR/ELISA healthy volunteers with and without menopause (n = 6 and 5, respectively).

The comparison of gene expression in qPCR/ELISA patients and healthy volunteers without menopause (n = 10 and 6, respectively) revealed a differential expression of five genes, namely *HMOX1* (*p* = 1.13 × 10^−6^), *KRT19* (*p* = 1.81 × 10^−4^), *HSPD1* (*p* = 9.00 × 10^−4^), *LDHA* (*p* = 0.047), and *MAPK1* (*p* = 0.004), as depicted in Figure 3. *HMOX1*, *KRT19*, *HSPD1*, and *MAPK1* were upregulated in patients compared to healthy volunteers, whereas *CA2* was downregulated. Considering the fact that the compared samples belonged to the individuals without menopause, we suggested that the observed changes in gene expression were caused by the disease. Similar results were obtained when we compared qPCR/ELISA patients and healthy volunteers with menopause (n = 10 and 5, respectively). In patients, changes in the expression of *HMOX1*, *KRT19*, *LDHA*, *HSPD1*, and *CA2* were significant (*p* < 0.05). *HMOX1*, *KRT19*, *LDHA*, and *HSPD1* were upregulated. The expression level of *MAPK1* did not change (*p* < 0.45) and *CA2* was downregulated.

In turn, the principle component analysis (PCA) of qPCR data revealed that a single factor (PC1) was responsible for 53% of the variability between the skin samples (Figure 3B). The K-mean clustering (Figure 3C) identified two clusters that contained samples of qPCR/ELISA psoriasis patients (n = 20) and healthy volunteers (n = 11). However, we could not completely separate patients with and without menopause, as well as similar groups of healthy volunteers.

A comparative analysis of gene expression in the PBMC obtained from the individuals that participated in the qPCR/ELISA experiments revealed significant changes in the expression of four genes, namely *HMOX1*, *HSPD1*, *LDHA*, and *KRT19*, whereas the expression *MAPK1* and *CA2* was not detected (Figure 3D). Similarly to skin cells, the expression levels of *HMOX1*, *HSPD1*, *LDHA*, and *KRT19* were higher in non-menopausal patients compared to menopausal patients. However, the changes in gene expression were statistically insignificant, except for *LDHA* (*p* = 0.047). Moreover, we did not see any significant changes in gene expression when we compared non-menopausal and menopausal volunteers.

In non-menopausal patients, the expression levels of *HMOX1*, *HSPD1*, *LDHA*, and *KRT19* were higher compared to non-menopausal volunteers (Figure 3D). In particular, we found that changes in the expression levels of *KRT19* and *HMOX1* were significant (*p* = 0.022 and 0.019, respectively), whereas changes in the expression levels of *HSPD1* and *LDHA* were statistically insignificant (*p* = 0.079 and 0.072, respectively). Similarly, the expression levels of *HMOX1*, *HSPD1*, *LDHA*, and *KRT19* were higher in menopausal patients compared to menopausal volunteers (Figure 3D). However, the changes in their expression levels were statistically insignificant (*p* = 0.0503 for *HMOX1*, *p* = 0.053 for *HSPD1*, *p* = 0.102 for *LDHA*, and *p* = 0.080 for *KRT19*).

### 3.4. Assessment of Protein Expression by ELISA Confirms a Differential Expression of KRT19 and HSPD1 in the Lesional Skin of Female Psoriasis Patients

**Figure 4:**
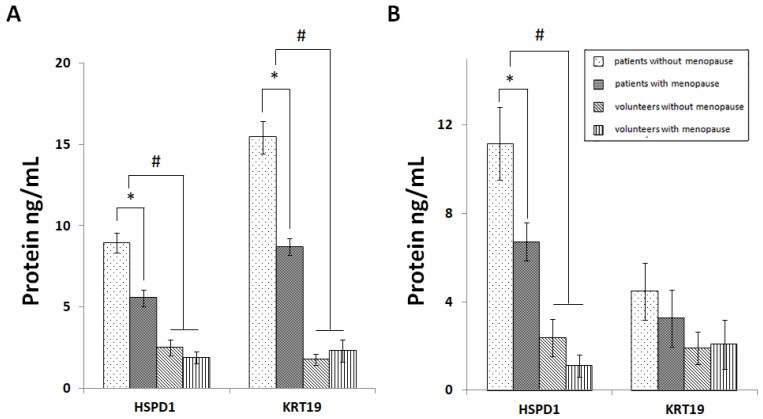
The levels of the expression of estrogen-responsive proteins in clinical samples of psoriasis patients and healthy volunteers, assessed by ELISA. (**A**). The levels of gene expression in the samples of patients’ lesional skin and in the skin of healthy volunteers. (**B**). The levels of gene expression in the PBMC obtained from the patients’ blood and from the blood of healthy volunteers. The following individuals participated in these experiments: psoriasis patients without menopause (n = 10); psoriasis patients with menopause (n = 10); healthy volunteers without menopause (n = 6); and healthy volunteers with menopause (n = 5). * *p* < 0.05 when women without menopause compared to women with menopause. ^#^ *p* < 0.05 when patients compared to healthy volunteers. Protein concentrations were measured in ng/mL.

Using ELISA, we analyzed the expression of KRT19 and HSPD1 in lesional skin as well as the PBMC of qPCR/ELISA psoriasis patients and healthy volunteers (Table 1). The performed analysis of skin samples revealed that the expression levels of both proteins were significantly higher in the lesional skin of patients compared to healthy volunteers (Figure 4A). Moreover, the expression levels of both proteins were significantly different (*p* < 0.05) in patients without menopause compared to patients with menopause. In contrast, we did not see significant differences in the expression of KRT19 and HSPD1 when we compared skin samples of non-menopausal and menopausal volunteers.

A similar analysis of the PBMC also revealed a significantly higher expression of HSPD1 in the lesional skin of patients compared to that of the healthy volunteers (Figure 4B). In contrast, changes in the expression of KRT19 were statistically insignificant although the expression level of KRT19 in patients’ PBMC was elevated compared to the PBMC of healthy volunteers. In a similar manner, the expression level of HSPD1 in the PBMC of the patients without menopause was significantly higher compared to the patients with menopause, although we did not see significant differences when we analyzed changes in the expression of KRT19. In addition, there were no significant differences in the expression of both proteins between non-menopausal and menopausal healthy volunteers.

## 4. Discussion

In this study, we analyzed skin samples of psoriasis patients using the LC–MS/MS method and reported of six ERPs, namely HMOX1, KRT19, LDHA, HSPD1, MAPK1, and CA2, that were differentially expressed in the lesional and uninvolved skin of female MS participants (Table 4) and were not present in the skin of their male counterparts (Table 5), suggesting an existence of a gender-dependent response to the disease. Using independent methods of analysis, namely qPCR and ELISA (Figure 3 and Figure 4), we examined their expression in menopausal and non-menopausal female patients and the respective groups of healthy volunteers (Figure 3A,D). We also assessed the levels of sex hormones, namely ES, PG, and TS, in their blood (Figure 1).

In performing qPCR and ELISA experiments on a larger cohort of women, we confirmed that the identified ERGs and their encoding proteins were differentially expressed in female lesional skin compared to healthy skin (Figure 3A and Figure 4A). In comparing non-menopausal and menopausal patients, we found significant changes in the expression of *HMOX1*, *HSPD1*, *CA2*, and *MAPK1*. In contrast, the changes in their expression were insignificant when we compared non-menopausal and menopausal healthy volunteers. The results of the ELISA experiments were similar to the results of the qPCR analysis (Figure 4). Thus, the obtained data suggested that the observed changes in gene expression were caused by the differences in the levels of sex hormones and were associated with the disease because healthy volunteers did not have them. As we believe, the sex hormones, primarily E2, acted as modulators, altering the expression of ERGs and interfering with the disease.

This hypothesis is in agreement with our next finding. In performing the PCA of qPCR data, we found that a single factor was responsible for 53% of the variability between the samples (Figure 3A). Using K-mean clustering, we separated the samples in two groups that contained samples of qPCR/ELISA patients and samples of qPCR/ELISA volunteers. However, we could not achieve complete separation of individuals with and without menopause within those groups. Based on this finding, we proposed that in some women, changes in the expression of ERGs might not coincide with menopause. As we believe, deviations of this kind could be caused by a crosstalk of ES-activated signaling mechanisms and other signaling pathways (e.g., the pathways activated by proinflammatory cytokines).

In assessing the levels of sex hormones in the blood of qPCR/ELISA participants, we showed (Figure 1) that the blood of patients contained significantly less E2 and PG, and more TS compared to that of the healthy control. Moreover, menopausal patients and healthy volunteers contained less E2 and PG, and more TS compared to their non-menopausal counterparts (Table 3). These results suggested that sex hormones influenced the gene expression in psoriatic skin. They also confirmed the previous findings [4,6,8] regarding that the fluctuations in the levels of E2, PG, and TS could potentially modulate the course of the disease.

In comparing gene expression profiles in lesional skin (Figure 3A) and the patterns of sex hormones in patients’ blood (Table 3), we discovered significant changes between menopausal and non-menopausal patients, as well as between healthy volunteers and any group of patients. The expression levels of *HMOX1*, *KRT19*, *LDHA*, *HSPD1*, and *MAPK1* were higher in patients with menopause compared to patients without menopause. Moreover, same individuals had higher blood levels of E2 and PG (Table 3). In addition, the expression levels of *CA2* and blood levels of TS were higher in patients with menopause compared to patients without menopause.

Furthermore, when we combined two groups of healthy volunteers with and without menopause (Figure 3A) and considered them as one group, we saw differences between the expression profiles of ERGs in lesional skin (Figure 3A) and in the patterns of sex hormones in their blood (Table 3). The expression levels of *HMOX1*, *KRT19*, *LDHA*, *HSPD1*, and *MAPK1* were lower in healthy volunteers compared to any group of patients. In contrast, their blood levels of E2 and PG were higher compared to the same groups. In contrast, the expression levels of *CA2* and blood levels of TS in both groups of patients were lower compared to that of healthy volunteers.

Based on these findings, we concluded that sex hormones differentially contributed to the expression of individual ERGs. Presumably, E2 and PG made a greater contribution to the regulation of *HMOX1*, *KRT19*, *LDHA*, *HSPD1*, and *MAPK1*, whereas TS made a greater contribution to the regulation of *CA2*. However, additional experimental studies on cultured cells are needed to clarify the role of particular sex hormones in the regulation of individual ERGs primarily in the skin because the biological effects of sex hormones are tissue-specific and their regulation of gene expression is often presented as a cross-talk of several signaling pathways.

In comparing the expression of the identified ERGs in the skin (Figure 3A) and of the PBMC (Figure 3D), we found similarities in their expression profiles. In particular, the expression levels of *HMOX1*, *KRT19*, *LDHA*, and *HSPD1* were significantly higher in the PBMC of patients compared to healthy volunteers, in non-menopausal patients compared to non-menopausal volunteers, and in menopausal patients compared to menopausal volunteers. However, most of these changes were statistically insignificant. Based on these findings, we concluded that sex hormones, primarily E2, influenced gene expression in both types of samples in a similar way. This result was anticipated since psoriasis is a systemic disorder that targets various tissues and is associated with multiple comorbidities. Expectedly, the same mechanism was activated in response to the disease in both the skin and PBMC.

The previous studies showed that female sex hormones have significant immunomodulatory effects. In particular, ES reduces the production of macrophage-attracting cytokines (CXCL8, CXCL10, CCL2, -5, and -8) and the production of IL12 in keratinocytes. These cytokines, namely CXCL10, CCL5, and -8, recruit activated T cells [17], macrophages [18], and neutrophils [19], respectively. E2 inhibits the production of IL12 and TNF by dendritic cells. Moreover, it decreases the blood level of neutrophils. Based on these findings, we may consider low E2 level in the blood of our patients (Figure 1) as a potential risk for exacerbation of the disease.

PG stimulates the production of the Th_2_ cytokines, namely IL4 and IL5, by T cells without altering the production of Th_1_ cytokines [20]. Moreover, PG blocks androgen receptors (AR) and, in inhibiting the release of LH, reduces the level of circulating androgens (AG) in the blood. In fibroblasts, PG suppresses the transcription of *CXCL8* [21]. In contrast, when the PG level is low, TS activates ARs, contributing to the development of an inflammatory response [22]. These data suggest that the reduction in the PG level in the patients’ blood that we observed in our study (Figure 1) could also contribute to the pathogenesis of the disease.

To our knowledge, this is the first study focused on gender-specific differences in gene and protein expression in psoriatic skin. Three of the six identified genes, namely *KRT19*, *LDHA*, and *HSPD1* were not previously associated with psoriasis. Depending on the role of the particular ERG in the pathogenesis of psoriasis, its differential expression may either promote or limit the growth of psoriatic plaques in a disease-affected area. Hemoxygenase 1 (HMOX1) is a stress protein. The expression of *HMOX1* can be induced by a variety of stimuli, including the proinflammatory cytokines TNF and IL17, which are abundant in the lesional skin of psoriasis patients [23]. The anti-inflammatory and anti-oxidant activities of HMOX1 are well-documented and can be considered as a part of a protective mechanism that contains (controls) the inflammatory response in lesional skin. The others already showed that inducers of *HMOX1* attenuate the inflammatory response in lesional skin [24]. Moreover, a proteolytic cleavage of HMOX1 generates several biologically active metabolites. One of them, the N-terminal peptide, binds to the promoter of *IL23A*, interfering with biological activities of proinflammatory cytokines IL12 and IL23 [25]. The others, bilirubin and CO, produce potent antioxidant and anti-inflammatory effects [26,27].

MAPK1 is one of two known extracellular signal-regulated kinases (ERKs). Although the role of MAPK1 in the inflammatory response, primarily for the induction of TNF [28], is well-documented, in a general sense, it is a signaling molecule that can be activated by multiple stimuli, including ES [29]. Due to the high sequence homology of ERKs and its similar role in the cell, two proteins, namely MAPK1/ERK2 and MAPK3/ERK1, are considered as two isoforms of the same enzyme. However, their expression patterns in lesional skin are different. MAPK3/ERK1 is strongly increased, whereas the expression of MAPK1/ERK2 is slightly affected by the disease [30]. The cited paper also suggests that, similarly to HMOX1, ES is likely to contribute to the regulation of MAPK1 in lesional skin. Particularly, the involvement of ES would explain the strong induction of *MAPK1* in our female patients and answer the question concerning why the expression of *MAPK1* significantly decreases after menopause in patients (Figure 3A).

We also discovered that *LDHA*, which encodes LDH-M, the subunit of lactate dehydrogenase (LDH), was upregulated in lesional skin (Figure 3A). Compared to the other LDH subunits, LDH-M has a higher affinity to pyruvate. In other words, if lactate and pyruvate are equally available, LDH-M preferentially binds to pyruvate and converts pyruvate to lactate. It also oxidizes NADH, which is a coenzyme in this reaction, to NAD^+^ [31]. According to the previously published data, *LDHA* is involved in several immunomodulatory processes that could potentially influence the flow of psoriasis. First, it decreases the proliferation of cytotoxic and other effector T cells and their production of cytokines because these cells become inactive at low glucose and high lactate concentrations in the medium [32]. Second, lactate activates a mechanism that stops the migration of T-cells, entrapping them in the inflamed area [33]. Third, a suppression of *LDHA* in macrophages reduces the phosphorylation of mitogen-activated protein kinase MAPK14/p38 [34]. At the same time, the accumulation of lactate in the inflamed tissue increases the production of IL17 by T cells [35]. In addition, *LDHA* is overexpressed in cancer cells that rely on aerobic glycolysis to maintain their higher proliferation and faster metabolic rates. For this reason, a higher expression of *LDHA* in lesional epidermis is also necessary due to hyperproliferation and acceleration of the metabolism in epidermal keratinocytes of psoriasis patients.

*HSPD1* encodes the mitochondrial chaperonin HSP60 that provides a favorable environment for the correct folding of unfolded and misfolded proteins. Similarly to *HMOX1*, *MAPK1*, and *LDHA*, *HSPD1* is induced in stress conditions. In cancer cells, a higher expression of *HSPD1* is needed due to their faster metabolic and proliferation rates, and more intensive protein trafficking between the cytoplasm and mitochondria [36]. Moreover, a higher expression of *HSPD1* in lesional skin, as we observed (Figure 3a), would help to maintain a faster turnover of the epidermal keratinocytes.

*KRT19* improves the rigidity of intermediate filaments [37]. Moreover, KRT19 activates NOTCH signaling pathways promoting nuclear translocation of β-catenin [38] and AKT [39]. The expression of *KRT19* in cancer cells increases their cell proliferation rate due to its ability to stabilize cyclin D3 [38]. A similar effect is observed in HaCaT cells that stop expressing *KRT19* before reaching the confluence [40]. We presume the upregulation of KRT19 in lesional skin helps epidermal keratinocytes to adapt to higher proliferation and metabolic rates in the areas affected by the disease.

*CA2*, which we also identified in our study, exhibits a higher catalytic rate for the conversion of bicarbonate to carbon dioxide compared to the other carbonic anhydrases. This prevents acidification of the cytoplasm. In the opposite direction, CA2 facilitates the diffusion of carbon dioxide through the cytoplasm, converting carbon dioxide to bicarbonate. According to our observations (Figure 3a), *CA2* levels were decreased in patients with menopause compared to either of the three groups, namely patients without menopause, healthy volunteers without menopause and healthy volunteers with menopause, suggesting that the downregulation of *CA2* in menopausal qPCR/ELISA patients (Figure 3a) could be caused by an action of an unidentified factor, such as TS which is capable of interfering with ES [41] and can be suppressed by PG [22].

**Figure 5:**
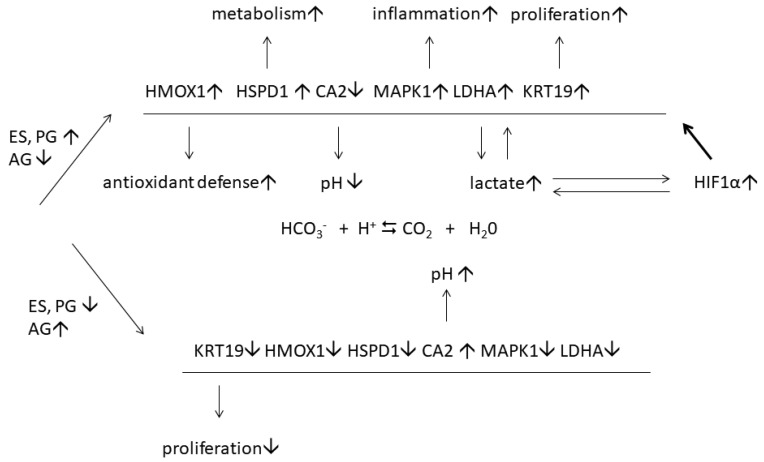
Regulation of estrogen-responsive proteins in lesional skin of female psoriasis patients with and without menopause: the proposed model. The signs ↑ and ↓ indicate that the protein expression is increased and decreased, respectively. The sign ⇆ indicates that chemical reaction between HCO_3_^−^ and can be reversed.

The differential expression of the identified ERGs can be part of an adaptive mechanism (Figure 5) that protects the skin cells from chronic inflammatory responses. Presumably, this mechanism is more efficient in female patients without menopause since they have higher levels of female sex hormones. Cooperating with several transcription factors, ES modulates the responses to various stimuli such as oxidative stress [42] and hypoxia [43]. For instance, there is an abundance of evidence regarding synergism between ES and hypoxia-induced factor 1α (HIF1α), which is highly unstable under normoxic conditions [44]. However, HIF1α becomes more stable in lesional skin [45] because lactate, the preferential product of LDH-M, stabilizes HIF1α by inhibiting prolyl hydroxylase (PHD2). It also triggers the nuclear translocation of HIF1α and the following induction of hypoxia responsive genes [46]. In contrast, the inhibition of LDHA causes a rapid degradation of HIF1α in proteasomes [47]. Many genes, including ones that we mentioned above, are common targets of ES and HIF1α [44,48]. Respectively, their expression (Figure 5) also depends on both ES and HIF1α [49]. It can be suppressed by either ERα antagonists [44] or inhibitors of ERα or HIF1α, as well as by HIF1α destabilizing factors.

The observed changes in the levels of sex hormones (Table 3) are of high clinical value. According to us (Figure 1) and others [22], low levels of sex hormones in the blood of women with psoriasis have a significant impact on the disease. Pregnant patients with moderate or severe forms of psoriasis have a higher risk of abortion, eclampsia, premature rupture of membranes, and macrosomia [50]. Another serious problem associated with psoriasis is female infertility [51]. However, it is still hard to say whether these complications are caused by psoriasis or comorbidities associated with the disease. For this reason, we explored the molecular basis of ES action in psoriatic skin and identified that there are six differentially expressed ERGs.

A medical correction of sex hormones may have a significant impact on the disease. The previously published data suggest that ES containing oral contraceptives [8] and hormone replacement therapy [52] may improve psoriasis. Conversely, it is well-documented that synthetic ES, if it is used as a therapeutic agent, can cause serious side effects such as cardiovascular events, thromboembolic disease, and breast cancer [53,54]. In this regard, knowledge of ERGs controlled by sex hormones in lesional skin suggests new hormone-free options to stabilize their expression in lesional skin, such as inducers of gene expression, biologically active peptides, and allosteric activators of their enzyme activity. Presumably, these potential treatment options may also help to avoid serious adverse effects.

In conclusion, a deficiency of female sex hormones seems to be among the risk factors that influence the flow of psoriasis in women. Respectively, the maintenance of their normal (physiological) levels may potentially prevent or suppress the disease. A detailed analysis of the proteins identified in the LC–MS/MS study, namely HMOX1, KRT19, LDHA, HSPD1, MAPK1, and CA2, revealed that they were differentially expressed in female lesional skin and were not present in male lesional skin. Using qPCR and ELISA, we found that the levels of their expression were higher in younger participants that were not in menopause. According to us and others, these proteins can be part of an adaptive mechanism that protects skin cells from the developing inflammatory response. In providing new insight on the molecular basis of ES signaling in female patients, we propose that ES mediates its anti-inflammatory effects in lesional skin, synergistically interacting with hormones/transcription factors that contribute to the stress response.

## Figures and Tables

**Table 1:** Clinical characteristics of psoriasis patients and volunteers that participated in the LC–MS/MS, qPCR, and ELISA studies.

ID	Age	Medical History	ID	Age	Medical History
		*LC-MS male patients*			*qPCR*/*ELISA patients*
1	30	not reported			*with menopause*
2	40	stage 1 arterial hyperten-	1	46	gastritis, cholecystitis,
		sion, hyperuricemia,			pancreatitis,
		obesity,			hypertension, coronary
3	68	psoriatic arthritis,			artery disease, angina
		hypertension, stage 2,			pectoris, urolithiasis
		bronchitis, dyscirculatory	2	46	hypertension, stage 2,
		encephalopathy,			kidney cyst
		hyperuricemia, obesity	3	47	cholecystitis,
		1st degree			cholelithiasis
			4	48	type II diabetes, obesity
		*LC-MS female patients*			stage 3,
1	48	stage 2 hypertension			arterial hypertension, no-
2	60	stage 2 hypertension			dular Hashimoto’s thy-
					roiditis, uterine fibroids,
		*LC-MS male volunteers*			cerebrovascular
1	53	phlebeurysm			disease, discirculatory
2	77	arthrosclerosis			encephalopathy arte-
					rial hypertension
		*LC-MS female volunteers*	5	51	tachycardia
1	39	white line hernia			nodular Hashimoto’s
2	60	incisional ventral hernia			thyroiditis,
3	79	arthrosclerosis, abdomi-			osteochondrosis
		nal aortic aneurysm,			dorsopathy
		chronic pyelonephritis,	6	51	hypertension, stage 2;
		dyslipidemia,	7	53	hypertension, stage 2;
		hypertension			depression;
			8	53	nodular goiter;
		*qPCR*/*ELISA patients,*	9	53	hypertension, stage 2
		*without menopause*			type 2 diabetes, osteo-
1	19	not reported			arthritis; dorsopathy;
2	21	erysipelas, obesity stage	10	54	not reported
		4, cholecystitis, uterine			*qPCR*/*ELISA volunteers*
		fibroids			*without menopause*
3	28	not reported	1	26	not reported
4	30	goiter, euthyroid sick	2	28	not reported
		syndrome,	3	29	not reported
5	32	bilateral otitis media,	4	32	not reported
		cholecystitis	5	33	not reported
		vaginal yeast infection	6	38	not reported
6	32	not reported			
7	32	diffuse goiter, grade 2;			*qPCR*/*ELISA volunteers*
8	36	depression			*with menopause*
9	42	gastritis, pyelonephritis;			
		insulin resistance,	1	43	not reported
		hypertension, stage 2,	2	49	not reported
		cerebrovascular disease,	3	50	not reported
		cholecystitis	4	55	not reported
10	43	not reported	5	57	not reported

**Table 2:** Gene-specific primers used in the qPCR experiments.

Gene	Reference Sequence	Primer Name	Primer Sequence	Product Size, bp
*CA2*	NM_000067.3	CA2 forward	GGCTGGTTGGTGCTTTGTTT	118
		CA2 re-verse	TTGTGAGTGCTCATCACCCT	
*HMOX1*	NM_002133.3	HMOX1 forward	GGCCTAAACTTCAGAGGGGG	99
		HMOX1 reverse	AGACAGCTGCCACATTAGGG	
*HSPD1*	NM_002156.5	HSPD1 forward	CTGGCACGCTCTATAGCCAA	142
		HSPD1 reverse	CAGGGGTGGTCACAGGTTTA	
*KRT19*	NM_002276.5	KRT19 forward	CCACTACTACACGACCATCCA	89
		KRT19 reverse	GTCGATCTGCAGGACAATCC	
*LDHA*	NM_005566.4	LDHA forward	TAAGCTGTCATGGGTGGGTC	100
		LDHA reverse	GGGTGCAGAGTCTTCAGAGAG	
*MAPK1*	NM_002745.5	MAPK1 forward	CAGTTCTTGACCCCTGGTCC	186
		MAPK1 reverse	TACATACTGCCGCAGGTCAC	
*18S RNA*	NR_003286.2	18S RNA forward	CTACCACATCCAAGGAAGCA	103
		18S RNA reverse	TTTTTCGTCACTACCTCCCCG	

**Table 3:** The levels of sex hormones in blood samples of qPCR/ELISA participants with and without menopause. pM and nM are concentrations (pmol/L and nmol/L, respectively).

Group of Participants	*N*	Estradiol, pM	Progesterone, nM	Testosterone, nM
Volunteers without	6	480.63 ± 157.43	8.18 ± 2.47	0.406 ± 0.078
menopause				
Volunteers with	5	53.8 ± 15.91	0.973 ± 0.206	0.733 ± 0.061
menopause				
*p*-value		0.026	0.018	0.018
Patients without	10	116.32 ± 45.85	0.159 ± 0.013	1.708 ± 0.362
menopause				
Patients with	10	14.51 ± 0.44	0.110 ± 0.011	3.428 ± 0.368
menopause				
*p*-value		0.0495	0.011	0.005

**Table 4:** The ontology analysis of proteins differentially expressed in female lesional and uninvolved skin. The analyzed proteins were differentially expressed in female lesional and uninvolved skin and were not differentially expressed in male lesional and uninvolved skincompared to the skin of healthy volunteers.

Term	Genes	*p*-Value	FDR
Translational initiation	RPL30, RPL10, RPS7, RPS8, RPL11, RPL13A, RPL23A, RPS15, RPS27, RPS19, RPL14, EIF3C, RPL28, EIF3D, EIF4G1, RPS12	8.59 × 10^−15^	6.62 × 10^−12^
Nuclear-transcribed mRNA catabolic process, nonsense- mediated decay	RPL30, RPL10, RPS7, RPS8, RPL11, RPL13A, RPL23A, RPS15, RPS27, RPS19, RPL14, RPL28, EIF4G1, RPS12	6.03 × 10^−^^13^	1.89 × 10^−10^
SRP-dependent cotranslational protein targeting to membrane	RPL30, RPL10, RPS7, RPS8, RPL11, RPL13A, RPL23A, RPS15, RPS27, RPS19, RPL14, RPL28, RPS12	7.37 × 10^−13^	1.89 × 10^−10^
Viral transcription	RPL30, RPL10, RPS7, RPS8, RPL11, RPL13A, RPL23A, RPS15, RPS27, RPS19, RPL14, RPL28, RPS12	6.20 × 10^−12^	1.19 × 10^−9^
Translation	RPL30, RPL10, RPS7, RPS8, RPL11, RPL13A, SARS, RPL23A, RPS15, RPS27, RPS19, RPL14, RPL28, EIF4G1, RPS12	7.59 × 10^−10^	1.17 × 10^−7^
rRNA processing	RPL30, RPL10, RPS7, RPS8, RPL11, RPL13A, DDX21, RPL23A, RPS15, RPS27, RPS19, RPL14, RPL28, RPS12	1.03 × 10^−9^	1.32 × 10^−7^
Cell-cell adhesion	LDHA, AHNAK, HSPA5, RPL14, TACSTD2, EFHD2, RPL23A, TAGLN2, ENO1, ALDOA, SPTBN2, EIF4G1	1.24 × 10^−6^	1.37 × 10^−4^
Regulation of mRNA stability	PSMD6, PSMD7, PSMD13, PSMC1, PSMD3, HSPB1, HSPA1B, EIF4G1	4.20 × 10^−6^	4.04 × 10^−4^
Response to estrogen	LDHA, KRT19, CA2, HMOX1, MAPK1, HSPD1	5.83 × 10^−5^	4.81 × 10^−3^
Glycolytic process	GPI, LDHA, PGAM1, ENO1, ALDOA	6.24 × 10^−5^	4.81 × 10^−3^
Regulation of cellular amino acid metabolic process	PSMD6, PSMD7, PSMD13, PSMC1, PSMD3	3.09 × 10^−4^	0.022
Canonical glycolysis	GPI, PGAM1, ENO1, ALDOA	5.88 × 10^−4^	0.038
Antigen processing and presentation of exogenous peptide antigen via MHC class I, TAP-dependent	PSMD6, PSMD7, PSMD13, PSMC1, PSMD3	6.95 × 10^−4^	0.041
NIK/NF-κB signaling	PSMD6, PSMD7, PSMD13, PSMC1, PSMD3	8.29 × 10^−4^	0.046

**Table 5:** The ontology analysis of proteins differentially expressed in male lesional and uninvolved skin. The analyzed proteins were differentially expressed in male lesional and uninvolved skin and were not differentially expressed in female lesional and uninvolved skin compared to the skin of healthy volunteers.

Term	Genes	*p*-Value	FDR
SRP-dependent cotran- slational protein targe- ting to membrane	RPS28, RPS16, RPL32, RPL23, RPL37A, RPL35A, FAU, RPL8, SRP14, RPL17, RPL19	4.74 × 10^−9^	4.95 × 10^−6^
Translational initiation	RPS28, RPS16, RPL32, EIF6, RPL23, RPL37A, RPL35A, FAU, RPL8, RPL17, RPL19	1.79 × 10^−7^	9.32 × 10^−5^
Viral transcription	RPS28, RPS16, RPL32, RPL23, RPL37A, RPL35A, FAU, RPL8, RPL17, RPL19	3.32 × 10^−7^	1.16 × 10^−4^
Nuclear-transcribed mRNA catabolic process, nonsense- mediated decay	RPS28, RPS16, RPL32, RPL23, RPL37A, RPL35A, FAU, RPL8, RPL17, RPL19	5.57 × 10^−7^	1.45 × 10^−4^
Translation	RPS28, RPS16, RPL32, RPL23, RPL37A, RPL35A, FAU, RPL8, RPL17, SLC25A6, RPL19	4.33 × 10^−5^	0.009
Negative regulation of endopeptidase activity	CSTB, CSTA, ITIH2, SERPIND1, SERPINF1, SERPINH1, SERPING1, COL6A3	6.01 × 10^−5^	0.010
rRNA processing	RPS28, RPS16, RPL32, RPL23, RPL37A, RPL35A, FAU, RPL8, RPL17, RPL19	6.56 × 10^−5^	0.010
Regulation of comple- ment activation	CFH, C9, C8B, PHB2, C8A	9.86 × 10^−5^	0.013
Complement activation alternative pathway	CFH, C9, C8B, C8A	1.43 × 10^−4^	0.017
Cell-cell adhesion	CNN2, PDLIM1, LAD1, DDX3X, ATIC, CTTN, RUVBL1, CHMP4B, PARK7, CNN3	3.86 × 10^−4^	0.040
DNA duplex unwinding	DDX3X, XRCC5, DDX1, RUVBL2, RUVBL1	4.47 × 10^−4^	0.042

## Data Availability

The mass spectrometry proteomics data were deposited to the ProteomeXchange Consortium via the PRIDE partner repository [15] with the dataset identifier PXD021673.

## Supplementary Materials

The following are available online at https://www.mdpi.com/article/10.3390/jpm11090925/s1, Table S1: Excel-file with heat map that describes changes in protein expression in the skin of the male and female individuals that participated in the LC–MS/MS study and the results of the statistical analysis in male (n = 3) and female psoriasis patients (n = 2) compared to healthy volunteers (n = 5). The differences in protein expression were analyzed using the Mann–Whitney U test.
