# Supplementary material for: Differential Expression of Estrogen-Responsive Genes in Women with Psoriasis
Source: J Pers Med. 2021 Sep 17;11(9):925. doi: 10.3390/jpm11090925 (PMC8465408; doi:10.3390/jpm11090925)
Supplement: Supplementary file 1 [file jpm-11-00925-s001.zip › Supplementary_table_S1_v6.pdf]

Table S1: Heat map that describes changes in protein expression in the skin of the male and female individuals that participated in the LC–MS/MS study and the results of the statistical analysis in male (n = 3) and female psoriasis patients (n = 2) compared to healthy volunteers (n = 5).
The differences in protein expression were analyzed using the Mann–Whitney U test.

min in raw — max in raw

Gene	Isoform	Uniprot ID	uninvolved vs. healthy skin	lesional vs. healthy skin	uninvolved vs. lesional skin	uninvolved female vs. healthy skin	lesional female vs healthy skin	uninvolved male vs. healthy skin	lesional male vs. healthy skin	DEPs, ♀ only	DEPs, ♂ only
A1BG		P04217	0.005	0.005	0.087	0.026	0.026	0.013	0.013		
A2M		P01023	0.087	0.038	0.458	0.123	0.123	0.051	0.148		
A2ML1		A8K2U0	0.301	0.005	0.008	0.219	0.026	0.013	0.441		
ABI3BP		Q7Z7G0	0.087	0.005	0.174	0.349	0.026	0.013	0.051		
ACAA2		P42765	0.232	0.024	0.232	0.349	0.219	0.013	0.090		♂
ACTN4		O43707	0.377	0.059	0.038	0.123	0.123	0.090	0.327		
ACTR2		P61160	0.174	0.024	0.005	0.123	0.026	0.090	0.327	♀	
ACTR3		A0A024RAI1	0.377	0.005	0.008	0.349	0.026	0.013	0.228		
ADH1B		P00325	0.125	0.087	0.059	0.500	0.026	0.327	0.051	♀	
ADIRF		Q15847	0.038	0.458	0.087	0.026	0.349	0.441	0.148	♀	
ADK	3	P55263-3	0.301	0.005	0.005	0.349	0.026	0.013	0.148		
AHCY		A0A384MT	0.005	0.005	0.005	0.026	0.026	0.013	0.013		
AHNAK		Q09666	0.024	0.024	0.232	0.061	0.026	0.090	0.051	♀	
AHNAK2	3	Q8IVF2-3	0.377	0.125	0.125	0.123	0.026	0.441	0.090	♀	
AIMP2		A8MU58	0.024	0.232	0.005	0.026	0.349	0.228	0.090	♀	
AK1		Q6FGX9	0.005	0.059	0.008	0.026	0.500	0.013	0.013		
AKR1B10		O60218	0.232	0.005	0.005	0.026	0.026	0.013	0.327		
ALB		F6KPG5	0.008	0.005	0.059	0.061	0.026	0.013	0.013		
ALDH1A1		P00352	0.232	0.038	0.125	0.219	0.061	0.090	0.051		
ALDH2		A0A384NPN	0.174	0.005	0.005	0.219	0.026	0.013	0.228		
ALDH3A1		P30838	0.024	0.087	0.125	0.061	0.349	0.013	0.051		♂
ALDH3A2	2	P51648-2	0.458	0.377	0.377	0.026	0.026	0.148	0.051	♀	
ALDH7A1	2	P49419-2	0.301	0.005	0.005	0.500	0.026	0.013	0.228		
ALDOA		P04075	0.458	0.024	0.024	0.349	0.026	0.090	0.441	♀	
ALDOC		A0A024QZ6	0.301	0.005	0.038	0.500	0.026	0.013	0.228		
ALYREF		Q86V81	0.458	0.008	0.024	0.123	0.026	0.026	0.148		
ANK1	Er13	P16157-14	0.024	0.125	0.024	0.061	0.219	0.148	0.051		
ANPEP		A0A024RC6	0.458	0.038	0.014	0.219	0.349	0.013	0.327		♂
Anti-FactorVIII scFv		A2KBC6	0.038	0.008	0.059	0.123	0.061	0.013	0.051		♂
ANXA1		Q5TZZ9	0.005	0.059	0.458	0.026	0.500	0.013	0.013		
ANXA11	2	P50995-2	0.059	0.377	0.038	0.123	0.349	0.441	0.090		
ANXA2		P07355	0.059	0.008	0.024	0.123	0.026	0.026	0.090		
ANXA8L1		Q5VT79	0.059	0.038	0.014	0.123	0.123	0.051	0.090		
AP2A2	2	O94973-2	0.301	0.174	0.014	0.349	0.219	0.228	0.327		
AP2B1	2	P63010-2	0.458	0.008	0.005	0.500	0.026	0.026	0.441		
AP2M1	2	Q96CW1-2	0.125	0.005	0.024	0.219	0.026	0.013	0.013		
APOA1		A0A024R3E	0.024	0.008	0.038	0.061	0.026	0.026	0.051		
APOA2		V9GYE3	0.087	0.005	0.059	0.219	0.026	0.013	0.090		
APOA4		P06727	0.087	0.008	0.038	0.349	0.061	0.013	0.051		♂
APOE		A0A0S2Z3D	0.014	0.005	0.038	0.061	0.026	0.013	0.026		
APOH		P02749	0.005	0.005	0.377	0.026	0.026	0.013	0.013		
ARCN1		B0YIW5	0.059	0.005	0.005	0.219	0.026	0.013	0.051		
ARF1		P84077	0.232	0.008	0.005	0.219	0.026	0.026	0.327		
ARG1		P05089	0.038	0.005	0.005	0.061	0.026	0.013	0.090		
ARHGAP1		Q07960	0.377	0.038	0.038	0.219	0.123	0.051	0.441		
ARHGDIA		P52565	0.014	0.301	0.014	0.123	0.349	0.327	0.013		♂
ARHGDIB		A0A024RAS	0.174	0.038	0.005	0.123	0.219	0.026	0.327		♂
ARPC1B		C9JTT6	0.377	0.008	0.005	0.219	0.061	0.013	0.441		♂
ARPC2		Q53R19	0.377	0.005	0.005	0.349	0.026	0.013	0.441		
ARPC3		Q2LE71	0.301	0.005	0.014	0.500	0.026	0.013	0.228		
ARPC4		P59998	0.377	0.024	0.005	0.219	0.061	0.051	0.441		
ARPC5		O15511	0.005	0.377	0.008	0.026	0.349	0.228	0.013		
ASPN		Q9BXN1	0.174	0.005	0.038	0.219	0.026	0.013	0.228		
ASPRV1		Q53RT3	0.232	0.005	0.059	0.500	0.026	0.013	0.148		
ATIC		P31939	0.301	0.014	0.014	0.219	0.061	0.026	0.441		♂
ATL3		Q6DD88	0.301	0.014	0.024	0.500	0.026	0.051	0.228	♀	
ATOX1		A0A2Z4T00	0.059	0.059	0.059	0.061	0.500	0.013	0.148		♂
ATP1A1	4	P05023-4	0.125	0.024	0.125	0.061	0.219	0.013	0.327		♂
ATP1B3		D3DNF9	0.377	0.038	0.038	0.349	0.026	0.148	0.441	♀	
ATP2A2	5	P16615-5	0.024	0.008	0.005	0.123	0.026	0.026	0.026		
ATP5F1A		P25705	0.174	0.038	0.008	0.349	0.123	0.051	0.148		
ATP5F1B		P06576	0.232	0.024	0.005	0.219	0.123	0.026	0.327		♂
ATP5F1C		Q8TAS0	0.301	0.087	0.024	0.349	0.349	0.051	0.327		
ATP5IF1		Q9UII2	0.024	0.005	0.125	0.219	0.026	0.013	0.013		
ATP5J		Q6IB54	0.377	0.059	0.005	0.349	0.123	0.090	0.441		
ATP5MF		P56134-4	0.014	0.125	0.377	0.026	0.219	0.148	0.051	♀	
ATP5MG		O75964	0.087	0.038	0.125	0.026	0.349	0.013	0.327		
ATP5PB		Q5QNZ2	0.232	0.008	0.014	0.219	0.061	0.013	0.327		♂
ATP6V1A		P38606	0.174	0.024	0.059	0.219	0.219	0.013	0.228		♂
ATP6V1B2		A0A140VK6	0.232	0.005	0.005	0.219	0.026	0.013	0.327		
ATP6V1E1		Q53Y06	0.174	0.014	0.014	0.349	0.026	0.051	0.148	♀	
AZGP1		P25311	0.014	0.005	0.024	0.123	0.026	0.013	0.013		
BASP1		P80723	0.377	0.232	0.038	0.500	0.500	0.148	0.327		
BCAP31		Q53HT6	0.458	0.024	0.005	0.349	0.061	0.051	0.327		
BGN		A6NLG9	0.059	0.038	0.174	0.026	0.026	0.148	0.228	♀	
BLVRB		P30043	0.014	0.174	0.014	0.061	0.219	0.228	0.026		♂
BPGM		A0A024R78	0.005	0.087	0.014	0.026	0.500	0.026	0.013		
BRWD1	1	Q59ER5	0.174	0.005	0.014	0.123	0.026	0.013	0.327		
BZW1	2	Q7L1Q6-2	0.005	0.014	0.005	0.026	0.026	0.051	0.013		
C10orf70		A0A024QZN	0.125	0.458	0.038	0.123	0.349	0.327	0.228		
C1QB		A0A0A0MS	0.087	0.038	0.458	0.349	0.123	0.051	0.051		
C1QBP		Q07021	0.458	0.005	0.014	0.349	0.026	0.013	0.441		
C1R		F5H2D0	0.087	0.038	0.087	0.061	0.061	0.090	0.228		
C1S		A0A087X23	0.024	0.014	0.125	0.061	0.061	0.026	0.051		♂
C2		A0A0G2JJM	0.014	0.008	0.232	0.123	0.026	0.026	0.013		
C3		P01024	0.005	0.005	0.377	0.026	0.026	0.013	0.013		
C6		H2PFF7	0.087	0.005	0.059	0.349	0.026	0.013	0.051		
C8A		P07357	0.087	0.014	0.174	0.500	0.061	0.026	0.026		♂
C8B		F5GY80	0.174	0.024	0.301	0.349	0.123	0.026	0.148		♂
C9		P02748	0.024	0.008	0.087	0.123	0.061	0.013	0.026		♂
C9orf88		A0A024R87	0.458	0.005	0.014	0.500	0.026	0.013	0.441		
CA1		P00915	0.014	0.125	0.008	0.061	0.123	0.228	0.026		♂
CA2		P00918	0.005	0.174	0.005	0.026	0.123	0.327	0.051	♀	
CALM1		B4DJ51	0.024	0.458	0.014	0.061	0.349	0.327	0.051		
CALML3		P27482	0.087	0.014	0.005	0.349	0.026	0.051	0.051	♀	
CALML5		Q9NZT1	0.301	0.005	0.005	0.123	0.026	0.013	0.051		
CALR		Q53G71	0.125	0.059	0.005	0.219	0.123	0.090	0.148		
CALU	3	O43852-3	0.232	0.059	0.005	0.123	0.123	0.090	0.441		
CAND1		Q86VP6	0.174	0.059	0.038	0.219	0.500	0.013	0.228		♂
CANX		P27824	0.458	0.024	0.059	0.500	0.026	0.090	0.441	♀	
CAP1		B2RDY9	0.458	0.014	0.005	0.500	0.061	0.026	0.441		♂
CAPN1		P07384	0.059	0.059	0.008	0.123	0.219	0.051	0.090		
CAPNS1		U3KQE2	0.087	0.038	0.125	0.026	0.349	0.013	0.327		
CAPZA1		P52907	0.458	0.038	0.005	0.349	0.123	0.051	0.441		
CAPZA2		Q53GE2	0.301	0.038	0.232	0.349	0.061	0.090	0.327		
CAPZB		B1AK87	0.301	0.087	0.024	0.349	0.219	0.090	0.327		
CAST	10	P20810-10	0.038	0.377	0.232	0.123	0.219	0.148	0.051		
CAVIN1		Q6NZI2	0.174	0.038	0.038	0.123	0.061	0.090	0.327		
CAVIN3		Q969G5	0.377	0.038	0.024	0.500	0.026	0.148	0.327	♀	
CBR1		P16152	0.174	0.087	0.377	0.349	0.500	0.026	0.051		♂
CBX3		A4D177	0.008	0.005	0.059	0.061	0.026	0.013	0.013		
CCT2		P78371	0.301	0.008	0.005	0.349	0.026	0.026	0.148		
CCT3		P49368	0.458	0.005	0.005	0.219	0.026	0.013	0.228		
CCT4		A8K3C3	0.024	0.005	0.005	0.123	0.026	0.013	0.026		
CCT5		P48643	0.301	0.005	0.005	0.219	0.026	0.013	0.441		
CCT6A		Q59ET3	0.458	0.005	0.005	0.500	0.026	0.013	0.441		
CCT7		Q6IBT3	0.125	0.005	0.005	0.123	0.026	0.013	0.228		
CCT8	theta	Q53HU0	0.458	0.005	0.005	0.349	0.026	0.013	0.441		
CDC37		A0A024R7B	0.087	0.005	0.008	0.500	0.026	0.013	0.026		
CDC42		A0A494BZX	0.377	0.059	0.005	0.349	0.123	0.090	0.441		
cDNA FLJ13913 fis		B3KN82	0.174	0.014	0.038	0.349	0.123	0.013	0.148		♂
cDNA FLJ16494 fis		B3KVF5	0.125	0.008	0.014	0.123	0.026	0.026	0.228		
cDNA FLJ34725		B3KRR1	0.174	0.087	0.059	0.123	0.349	0.013	0.327		♂
cDNA FLJ46506 fis		B3KY04	0.038	0.232	0.059	0.219	0.349	0.090	0.026		♂
cDNA FLJ52561		B7Z4B7	0.087	0.014	0.087	0.026	0.061	0.026	0.327		
cDNA FLJ52869		B7Z4K8	0.087	0.038	0.038	0.500	0.219	0.026	0.026		♂
cDNA FLJ53075		B4DPP8	0.038	0.008	0.038	0.061	0.026	0.026	0.090		
cDNA FLJ53422		B4E312	0.125	0.005	0.014	0.123	0.026	0.013	0.228		
cDNA FLJ53910		B4DRR0	0.125	0.005	0.005	0.123	0.026	0.013	0.228		
cDNA FLJ54622		B4DDT3	0.024	0.005	0.087	0.123	0.026	0.013	0.026		
cDNA FLJ56425		B4DEA8	0.174	0.174	0.014	0.123	0.219	0.228	0.327		
cDNA FLJ56954		B7Z539	0.024	0.059	0.232	0.123	0.500	0.013	0.026		♂
cDNA FLJ57740		B4DKN9	0.059	0.059	0.008	0.123	0.123	0.090	0.090		
cDNA FLJ61158		B4DQT8	0.458	0.059	0.038	0.349	0.500	0.013	0.441		♂
cDNA FLJ76817		A8K525	0.458	0.014	0.005	0.349	0.061	0.026	0.327		♂
cDNA, FLJ79477		B0AZN7	0.377	0.005	0.005	0.123	0.026	0.013	0.090		
cDNA, FLJ93657		B2R7Y3	0.377	0.008	0.008	0.219	0.061	0.013	0.148		♂
cDNA, FLJ93711		B2R829	0.059	0.125	0.008	0.349	0.061	0.327	0.026		♂
cDNA, FLJ94025		B2R8R5	0.377	0.014	0.008	0.349	0.061	0.026	0.441		♂
cDNA, FLJ96345		B2RCX0	0.087	0.014	0.008	0.219	0.061	0.051	0.090		
CES1	3	P23141-3	0.038	0.008	0.174	0.061	0.026	0.026	0.090		
CFH		P08603	0.008	0.024	0.301	0.061	0.061	0.051	0.013		♂
CFL1		P23528	0.059	0.005	0.005	0.219	0.026	0.013	0.051		
CHMP4B		Q9H444	0.087	0.024	0.174	0.123	0.219	0.013	0.148		♂
CIRBP		Q53XX5	0.232	0.301	0.024	0.219	0.219	0.441	0.327		
CKAP4		Q8TB01	0.024	0.038	0.005	0.123	0.061	0.090	0.026		♂
CKB		P12277	0.301	0.024	0.038	0.219	0.219	0.013	0.090		♂
CKMT1A		P12532	0.008	0.005	0.005	0.061	0.026	0.013	0.013		
CLIC1		Q5SRT3	0.301	0.087	0.024	0.500	0.123	0.148	0.228		
CLIC3		Q6FGP1	0.087	0.005	0.005	0.061	0.026	0.013	0.228		
CLTC	2	Q00610-2	0.301	0.005	0.005	0.500	0.026	0.013	0.228		
CLU	variant 4	P10909-4	0.059	0.024	0.232	0.061	0.061	0.051	0.148		
CMA1		P23946	0.301	0.008	0.008	0.061	0.026	0.026	0.327		
CMPK1		P30085	0.377	0.059	0.014	0.349	0.219	0.051	0.441		
CNDP2		Q96KP4	0.377	0.014	0.014	0.349	0.061	0.026	0.441		♂
CNN1		P51911	0.038	0.458	0.059	0.061	0.349	0.441	0.090		
CNN2		B4DUT8	0.014	0.125	0.005	0.061	0.349	0.090	0.026		♂
CNN3	3	Q15417-3	0.301	0.014	0.087	0.123	0.123	0.013	0.441		♂
COL12A1		A0A087X0A	0.005	0.024	0.125	0.026	0.026	0.090	0.013		
COL14A1	2	Q05707-2	0.014	0.038	0.377	0.061	0.026	0.148	0.026		
COL1A1	CRA_a	D3DTX7	0.059	0.014	0.125	0.500	0.123	0.013	0.013		
COL1A1		P02452	0.087	0.005	0.087	0.500	0.026	0.013	0.026		♂
COL1A2		A0A087WTA	0.059	0.005	0.087	0.500	0.026	0.013	0.013		
COL6A1		A0A087X0S	0.125	0.014	0.024	0.123	0.026	0.051	0.228	♀	
COL6A3		D9ZGF2	0.301	0.014	0.024	0.349	0.026	0.051	0.327	♀	
COL6A3	4	P12111-4	0.232	0.008	0.059	0.349	0.061	0.013	0.228		♂
COMT	soluble	P21964-2	0.377	0.038	0.038	0.123	0.219	0.026	0.327		♂
COPA		A0A3B3IT15	0.377	0.024	0.014	0.349	0.123	0.026	0.441		♂
COPE		Q53HJ6	0.301	0.008	0.005	0.349	0.026	0.026	0.327		
COPG1		Q9Y678	0.038	0.024	0.014	0.123	0.219	0.013	0.051		♂
CORO1A		A0A024R61	0.014	0.458	0.005	0.026	0.500	0.441	0.051	♀	
CORO1C	3	Q9ULV4-3	0.038	0.232	0.458	0.123	0.500	0.148	0.051		
COTL1		Q14019	0.377	0.008	0.008	0.349	0.061	0.013	0.441		♂
COX2		H9STE0	0.232	0.458	0.125	0.123	0.500	0.441	0.026		♂
COX4I1		P13073	0.232	0.005	0.005	0.349	0.026	0.013	0.228		
COX5B		P10606	0.301	0.005	0.005	0.349	0.026	0.013	0.327		
COX6A1		Q9UEG9	0.458	0.024	0.087	0.349	0.219	0.013	0.327		♂
CP		A5PL27	0.005	0.008	0.301	0.026	0.026	0.026	0.013		
CRABP2		P29373	0.174	0.005	0.005	0.349	0.026	0.013	0.148		
CRIP2		Q53FN1	0.038	0.059	0.024	0.123	0.123	0.090	0.051		
CRYAB		P02511	0.458	0.087	0.014	0.500	0.123	0.148	0.441		
CRYZ		Q08257	0.125	0.087	0.377	0.026	0.026	0.327	0.441	♀	
CS		O75390	0.458	0.059	0.008	0.500	0.123	0.090	0.441		
CSNK1A1	2	P48729-2	0.377	0.014	0.014	0.219	0.123	0.013	0.441		♂
CSTA		P01040	0.377	0.024	0.024	0.219	0.219	0.013	0.441		♂
CSTB		Q76LA1	0.059	0.008	0.005	0.123	0.061	0.013	0.090		♂
CTNNA1	2	P35221-2	0.458	0.059	0.038	0.123	0.219	0.051	0.228		
CTSB		P07858	0.458	0.059	0.038	0.500	0.500	0.013	0.441		♂
CTSD		P07339	0.059	0.005	0.014	0.123	0.026	0.013	0.090		
CTSG		P08311	0.125	0.024	0.059	0.061	0.061	0.051	0.327		
CTTN		Q53HG7	0.174	0.014	0.087	0.349	0.061	0.026	0.148		♂
CYB5R1		Q9UHQ9	0.059	0.301	0.301	0.349	0.349	0.148	0.026		♂
CYBRD1	3	Q53TN4-3	0.059	0.174	0.301	0.026	0.219	0.228	0.228	♀	
CYCS		C9JFR7	0.174	0.005	0.038	0.026	0.026	0.013	0.441		
DARS1	cytoplasmic	A0A140VJW	0.174	0.038	0.087	0.349	0.123	0.051	0.051		
DBI		P07108	0.301	0.024	0.024	0.349	0.219	0.013	0.327		♂
DBNL	2	Q9UJU6-2	0.458	0.059	0.008	0.349	0.061	0.148	0.441		
DCD		P81605	0.059	0.458	0.125	0.026	0.219	0.228	0.228	♀	
DCN		Q6FH10	0.458	0.024	0.005	0.349	0.061	0.051	0.441		
DCXR		A0A384NY1	0.024	0.232	0.059	0.061	0.349	0.090	0.051		

			volunteers					patients																		
			females			males		uninvolved					lesional													
								females		males			females		males											
Gene	Isoform	Uniprot ID	FH1	FH2	FH3	FH4	FH5	FU1	FU2	MU1	MU2	MU3	FL1	FL2	ML1	ML2	ML3	uninvolved vs. healthy skin	lesional vs. healthy skin	uninvolved vs. lesional skin	uninvolved female vs. healthy skin	lesional female vs healthy skin	uninvolved male vs. healthy skin	lesional male vs. healthy skin	DEPs, ♀ only	DEPs, ♂ only
DDAH2		O95865																0.005	0.005	0.024	0.026	0.026	0.013	0.013		
DDOST	3	P39656-3																0.232	0.059	0.059	0.123	0.500	0.013	0.441		
DDX1	3	Q92499-3																0.232	0.059	0.024	0.349	0.349	0.026	0.228		♂
DDX21		Q9NR30																0.377	0.014	0.014	0.500	0.026	0.051	0.327	♀	
DDX39B	2	Q13838-2																0.458	0.005	0.005	0.500	0.026	0.013	0.441		
DDX3X		A0A0D9SF5																0.458	0.014	0.014	0.219	0.123	0.013	0.327		♂
DDX48		A0A024R8W																0.301	0.008	0.005	0.349	0.026	0.026	0.327		
DDX5		P17844																0.458	0.005	0.005	0.500	0.026	0.013	0.441		
DEK,		P35659-2																0.059	0.005	0.125	0.500	0.026	0.013	0.013		
DES		P17661																0.024	0.377	0.038	0.061	0.219	0.441	0.051		
DF		Q6FHW3																0.087	0.008	0.008	0.349	0.026	0.026	0.051		
DHX9		Q08211																0.087	0.005	0.014	0.026	0.026	0.013	0.327		
DLST	2	P36957-2																0.059	0.059	0.377	0.349	0.123	0.090	0.026		♂
DMKN	5	Q6E0U4-16																0.087	0.458	0.377	0.500	0.061	0.148	0.026		♂
DNAJB1		Q6FI51																0.232	0.008	0.014	0.500	0.026	0.013	0.148		
DNCL1		Q6FGH9																0.174	0.059	0.377	0.219	0.123	0.090	0.026		♂
DPYSL2		Q16555																0.059	0.014	0.059	0.123	0.026	0.051	0.090	♀	
DPYSL3		Q8IXW6																0.005	0.005	0.005	0.026	0.026	0.013	0.013		
DRIP4		Q4W4Y1																0.038	0.301	0.014	0.123	0.349	0.327	0.051		
DSG1		Q02413																0.458	0.174	0.174	0.123	0.026	0.441	0.148	♀	
DSP		P15924																0.174	0.174	0.174	0.026	0.026	0.441	0.441	♀	
DST	3	Q03001-3																0.174	0.301	0.087	0.500	0.026	0.228	0.090	♀	
DYNC1H1		Q14204																0.458	0.014	0.014	0.123	0.123	0.013	0.228		♂
ECH1		Q13011																0.059	0.038	0.458	0.123	0.123	0.051	0.090		
ECHS1		A0A384MDY																0.458	0.059	0.038	0.349	0.500	0.013	0.441		♂
EEA1		Q15075																0.232	0.038	0.038	0.500	0.219	0.026	0.148		♂
EEF1A1		Q6IPS9																0.458	0.008	0.005	0.500	0.061	0.013	0.441		♂
EEF1B2		A0A024R3W																0.301	0.005	0.005	0.500	0.026	0.013	0.228		
EEF1D		P29692																0.458	0.008	0.005	0.500	0.026	0.026	0.441		
EEF1G		Q53YD7																0.125	0.005	0.005	0.123	0.026	0.013	0.228		
EEF2		P13639																0.125	0.005	0.005	0.349	0.026	0.013	0.090		
EFHD2		A0A024QZ7																0.024	0.125	0.014	0.026	0.219	0.148	0.090	♀	
EHD1		B2R5U3																0.059	0.087	0.005	0.219	0.061	0.228	0.051		
EIF2S1		Q53XC0																0.174	0.005	0.005	0.349	0.026	0.013	0.148		
EIF2S3		P41091																0.377	0.005	0.005	0.349	0.026	0.013	0.441		
EIF3A		Q24JU4																0.458	0.005	0.005	0.349	0.026	0.013	0.441		
EIF3B		Q96G38																0.059	0.458	0.024	0.123	0.219	0.327	0.090		
EIF3C		B4DVQ5																0.174	0.005	0.005	0.349	0.026	0.013	0.148		
EIF3D	2	O15371-2																0.458	0.038	0.005	0.349	0.026	0.148	0.441	♀	
EIF3F		B4DMT5																0.377	0.005	0.005	0.349	0.026	0.013	0.228		
EIF3L	2	Q9Y262-2																0.174	0.005	0.005	0.219	0.026	0.013	0.228		
EIF3M		Q7L2H7																0.232	0.038	0.005	0.500	0.123	0.051	0.148		
EIF3S2		Q5U0F4																0.458	0.005	0.005	0.500	0.026	0.013	0.441		
EIF4A1		P60842																0.087	0.005	0.005	0.219	0.026	0.013	0.090		
EIF4E		Q32Q75																0.301	0.005	0.038	0.219	0.026	0.013	0.090		
EIF4G1		B2RU10																0.174	0.059	0.038	0.061	0.026	0.228	0.441	♀	
EIF4H	short	Q15056-2																0.232	0.005	0.005	0.061	0.026	0.013	0.441		
EIF5A		I3L397																0.458	0.024	0.014	0.500	0.219	0.013	0.441		♂
EIF5B		Q8N5A0																0.038	0.014	0.008	0.349	0.026	0.051	0.013		
EIF6		P56537																0.125	0.024	0.005	0.219	0.123	0.026	0.148		♂
EL52		K9JA46																0.174	0.008	0.005	0.123	0.026	0.026	0.327		
ENO1		P06733																0.174	0.024	0.005	0.349	0.026	0.090	0.148	♀	
EPB41		P11171																0.125	0.458	0.024	0.123	0.500	0.441	0.228		
EPB42	3	P16452-3																0.008	0.038	0.024	0.026	0.123	0.051	0.026		
EPPK1		P58107																0.232	0.377	0.174	0.349	0.123	0.327	0.090		
EPRS1		P07814																0.458	0.005	0.005	0.349	0.026	0.013	0.327		
ERH		P84090																0.232	0.059	0.059	0.349	0.349	0.026	0.090		♂
ERP29		P30040																0.174	0.059	0.005	0.123	0.123	0.090	0.327		
ERP44		Q9BS26																0.301	0.059	0.014	0.219	0.219	0.051	0.441		
ETF1		P62495																0.174	0.005	0.005	0.500	0.026	0.013	0.090		
ETFA	2	P13804-2																0.458	0.232	0.038	0.500	0.349	0.228	0.441		
EVPL		A0A024R8L																0.377	0.174	0.174	0.219	0.349	0.051	0.148		
EZR		P15311																0.377	0.005	0.008	0.026	0.026	0.013	0.026		
FABP4		E7DVW4																0.038	0.008	0.458	0.061	0.061	0.013	0.090		♂
FABP5		Q01469																0.059	0.005	0.005	0.123	0.026	0.013	0.090		
FAM162A		F8W7Q4																0.232	0.059	0.024	0.500	0.026	0.228	0.148	♀	
FAM83H		Q6ZRV2																0.458	0.038	0.087	0.123	0.123	0.051	0.228		
FASN		P49327																0.377	0.458	0.301	0.349	0.500	0.441	0.441		
FAU		P62861																0.377	0.125	0.174	0.349	0.349	0.026	0.228		♂
FBLN1		B4DUV1																0.024	0.458	0.038	0.026	0.500	0.441	0.090	♀	
FBLN2		Q86V58																0.038	0.005	0.005	0.123	0.026	0.013	0.051		
FGA	2	P02671-2																0.014	0.059	0.458	0.026	0.219	0.051	0.051	♀	
FH	cytoplasmic	P07954-2																0.377	0.014	0.008	0.349	0.061	0.026	0.228		♂
FKBP3		Q00688																0.174	0.008	0.024	0.219	0.061	0.013	0.228		♂
FKBP4		Q02790																0.232	0.125	0.014	0.219	0.123	0.228	0.327		
FLG	predicted	Q59H78																0.059	0.008	0.232	0.026	0.026	0.026	0.228		
FLG		P20930																0.038	0.014	0.087	0.026	0.026	0.051	0.148	♀	
FLG2		Q5D862																0.125	0.005	0.059	0.026	0.026	0.013	0.441		
FLNA	2	P21333-2																0.125	0.377	0.232	0.061	0.500	0.327	0.327		
FLNB	2	O75369-2																0.301	0.232	0.377	0.349	0.061	0.441	0.327		
FLNC	2	Q14315-2																0.125	0.458	0.377	0.026	0.219	0.327	0.441	♀	
FN1		A0A024R46																0.024	0.024	0.301	0.061	0.123	0.026	0.051		♂
FSCN1		A0A384ME																0.458	0.059	0.005	0.500	0.123	0.090	0.441		
FTCD		O95954																0.008	0.458	0.005	0.026	0.500	0.441	0.026		
FTL		A0A384MD																0.005	0.005	0.174	0.026	0.026	0.013	0.013		
G3BP1		Q13283																0.174	0.005	0.005	0.219	0.026	0.013	0.228		
G6PD	long	P11413-2																0.232	0.024	0.024	0.349	0.026	0.090	0.228	♀	
GANAB		Q14697																0.014	0.174	0.005	0.061	0.349	0.148	0.026		♂
GAPDH		P04406																0.301	0.059	0.005	0.349	0.123	0.090	0.327		
GARS		A0A090N8																0.038	0.005	0.005	0.349	0.026	0.013	0.013		
GC		P02774																0.005	0.005	0.014	0.026	0.026	0.013	0.013		
GCT-A5 light chain variab		A0A0X9UW																0.014	0.014	0.458	0.061	0.061	0.026	0.026		♂
GDI2		Q6IAT1																0.458	0.005	0.005	0.500	0.026	0.013	0.441		
GFUS		Q13630																0.174	0.008	0.059	0.349	0.026	0.026	0.148		
GGCT		O75223																0.125	0.005	0.005	0.123	0.026	0.013	0.228		
GGH		Q92820																0.125	0.059	0.024	0.061	0.500	0.013	0.327		♂
GIB6		A0A024RDS																0.301	0.087	0.038	0.349	0.123	0.148	0.327		
GLTP		F5H0U5																0.301	0.008	0.005	0.500	0.026	0.026	0.327		
GM2A		P17900																0.232	0.038	0.059	0.219	0.349	0.013	0.327		♂
GNAI2	sGi2	P04899-4																0.005	0.377	0.005	0.026	0.349	0.228	0.013		
GNB1		P62873																0.038	0.301	0.301	0.123	0.219	0.441	0.051		
GOT1		P17174																0.301	0.059	0.024	0.123	0.061	0.148	0.441		
GOT2	2	P00505																0.087	0.008	0.038	0.061	0.061	0.013	0.228		♂
GPI		A0A1W6AY																0.087	0.014	0.005	0.123	0.026	0.051	0.148	♀	
GSDMA		Q96QA5																0.125	0.005	0.005	0.219	0.026	0.013	0.148		
GSN		P06396																0.125	0.232	0.024	0.349	0.500	0.148	0.090		
GSN	2	P06396-2																0.087	0.005	0.005	0.219	0.026	0.013	0.090		
GSTO1		P78417																0.125	0.008	0.014	0.026	0.026	0.026	0.441		
GSTP1		P09211																0.458	0.005	0.024	0.349	0.026	0.013	0.441		
H1-0		P07305																0.232	0.059	0.059	0.349	0.026	0.228	0.228	♀	
H1-3		P16402																0.087	0.125	0.024	0.123	0.500	0.051	0.148		
H1-5		P16401																0.125	0.301	0.232	0.026	0.219	0.090	0.441	♀	
H2AC21		Q8IUE6																0.174	0.008	0.005	0.026	0.500	0.026	0.090		
H2AZ2		Q71UI9																0.125	0.125	0.377	0.026	0.061	0.327	0.441	♀	
H2BC10		P62807																0.458	0.005	0.005	0.349	0.026	0.013	0.327		
H2BC21		Q16778																0.232	0.008	0.014	0.123	0.026	0.013	0.441		
H3F3A		B4E380																0.038	0.024	0.377	0.061	0.123	0.026	0.090		♂
HARS1		P12081																0.377	0.005	0.005	0.349	0.026	0.013	0.441		
HBA2	mutant	Q4ZGM8																0.377	0.005	0.174	0.349	0.026	0.013	0.441		
HBA2		A0A1S5UZ3																0.008	0.458	0.008	0.026	0.349	0.441	0.026		
HBB		D9YZU5																0.008	0.377	0.005	0.026	0.349	0.441	0.026		
HBD		A0N071																0.038	0.174	0.087	0.123	0.219	0.228	0.051		
HDGF		P51858																0.125	0.005	0.038	0.219	0.026	0.013	0.148		
HIST1H1E		A3R0T8																0.458	0.014	0.024	0.219	0.026	0.051	0.327	♀	
HIST1H2AC		A0A024R01																0.232	0.174	0.024	0.500	0.123	0.327	0.148		
HIST1H2AK		A4FTV9																0.458	0.059	0.014	0.219	0.123	0.090	0.327		
HK1	3	P19367-3																0.125	0.008	0.024	0.123	0.026	0.026	0.228		
HLA-A		A0A0U5PYZ																0.174	0.005	0.024	0.123	0.026	0.013	0.327		
HLA-B		C7FDR3																0.458	0.087	0.038	0.219	0.061	0.228	0.327		
HMGB2		P26583																0.377	0.059	0.024	0.219	0.219	0.051	0.441		
HMGN4		A0A024R04																0.125	0.301	0.038	0.500	0.500	0.228	0.051		
HMOX1		Q6FH11																0.377	0.014	0.008	0.349	0.026	0.051	0.441	♀	
HNRNPA0		Q13151																0.024	0.024	0.005	0.026	0.061	0.051	0.090	♀	
HNRNPA1		P09651																0.377	0.005	0.005	0.219	0.026	0.013	0.441		
HNRNPA2B1		P22626																0.377	0.024	0.008	0.219	0.219	0.013	0.441		♂
HNRNPA3	2	P51991-2																0.232	0.059	0.024	0.219	0.123	0.090	0.327		
HNRNPAB		D6R9P3																0.087	0.038	0.038	0.500	0.026	0.026	0.026		
HNRNPC		G3V4W0																0.038	0.005	0.005	0.123	0.026	0.013	0.051		
HNRNPD		H0Y8G5																0.174	0.005	0.005	0.026	0.026	0.013	0.441		
HNRNPDL	3	O14979-3																0.174	0.125	0.014	0.219	0.123	0.228	0.228		
HNRNPH1		G8JLB6																0.059	0.024	0.005	0.061	0.061	0.051	0.148		
HNRNPH3	2	P31942-2																0.232	0.038	0.174	0.219	0.026	0.148	0.327	♀	
HNRNPK	3	P61978-3																0.377	0.005	0.005	0.500	0.026	0.013	0.327		
HNRNPL		Q6NTA2																0.377	0.005	0.005	0.349	0.026	0.013	0.441		
HNRNPM		P52272																0.458	0.008	0.005	0.500	0.026	0.026	0.441		
HNRNPR		O43390																0.377	0.038	0.005	0.349	0.123	0.051	0.228		
HNRNPU		Q00839																0.174	0.005	0.014	0.123	0.026	0.013	0.327		
HNRNPUL2		Q1KMD3																0.232	0.059	0.059	0.219	0.349	0.026	0.327		♂
HNRPA3		A0A384NL6																0.125	0.125	0.014	0.219	0.349	0.090	0.148		
HNRPF		A0A024R7T																0.301	0.008	0.005	0.219	0.026	0.026	0.090		
HP		P00738																0.014	0.014	0.458	0.026	0.061	0.026	0.051		
HPX		P02790																0.024	0.008	0.232	0.061	0.061	0.013	0.051		♂
HRG		P04196																0.005	0.005	0.232	0.026	0.026	0.013	0.013		
HSD17B12		B4DWS6																0.377	0.014	0.014	0.500	0.026	0.051	0.327	♀	
HSD17B4		P51659																0.301	0.059	0.059	0.219	0.500	0.013	0.090		♂
HSP70B		Q53FC7																0.125	0.087	0.059	0.123	0.500	0.026	0.228		♂
HSP90AB1		A0A024RD8																0.458	0.005	0.005	0.500	0.026	0.013	0.441		
HSPA1B		A8K5I0																0.059	0.014	0.125	0.123	0.026	0.051	0.090	♀	
HSPA4		P34932																0.377	0.008	0.008	0.349	0.026	0.026	0.228		
HSPA4L		E9PDE8																0.014	0.038	0.125	0.026	0.061	0.090	0.051	♀	
HSPA5		P11021																0.087	0.014	0.005	0.123	0.026	0.051	0.148	♀	
HSPA8		P11142																0.174	0.005	0.005	0.219	0.026	0.013	0.228		
HSPA9		P38646																0.059	0.014	0.014	0.123	0.061	0.026	0.090		♂
HSPB1		V9HW43																0.087	0.458	0.059	0.026	0.500	0.441	0.327	♀	
HSPB6		A0A1X7SC6																0.038	0.008	0.038	0.123	0.061	0.013	0.051		♂
HSPD1		P10809																0.301	0.008	0.005	0.026	0.026	0.441	0.228	♀	
HSPE1		P61604																0.458	0.014	0.005	0.500	0.061	0.026	0.441		♂
HSPG2		A0A024RAB																0.232	0.024	0.087	0.349	0.061	0.051	0.228		
HYOU1		A0A494C03																0.125	0.014	0.005	0.123	0.026	0.026	0.228		
IARS1		P41252																0.301	0.005	0.005	0.500	0.026	0.013	0.228		
IBM-B2 heavy chain varia		A0A125QYY																0.038	0.014	0.301	0.349	0.061	0.013	0.013		♂
IDE		A0A3B3IGS																0.458	0.005	0.005	0.219	0.026	0.013	0.327		
IDH3A		B4DJ84																0.301	0.087	0.038	0.349	0.061	0.228	0.327		
IFI16		D3DUZ3																0.125	0.005	0.005	0.349	0.026	0.013	0.148		
IgG H chain		S6B291																0.125	0.038	0.125	0.349	0.061	0.090	0.090		
IGHV3-15		A0A0B4J1V																0.014	0.038	0.059	0.123	0.123	0.051	0.013		♂
IGK@		Q6PJF2																0.008	0.005	0.087	0.061	0.026	0.013	0.013		
IGL@		Q6N595																0.014	0.038	0.458	0.061	0.219	0.026	0.026		♂
IGL@		Q8N355																0.125	0.024	0.059	0.349	0.123	0.026	0.090		♂
IGv domain-containing pr		B7ZMG8																0.008	0.008	0.174	0.061	0.061	0.441	0.327		
IL1RN	2	P18510-2																0.087	0.005	0.008	0.349	0.026	0.013	0.051		
IL36G		Q9NZH8																0.125	0.014	0.005	0.349	0.026	0.051	0.090	♀	
IL36RN		C9JTH1																0.174	0.024	0.008	0.123	0.026	0.090	0.327	♀	
ILF2		B4DY09																0.301	0.014	0.014	0.500	0.123	0.013	0.228		♂
ILF3	7	Q12906-7																0.125	0.014	0.005	0.123	0.026	0.051	0.228	♀	
Immunglobulin heavy cha		Q0ZCH9																0.014	0.014	0.087	0.123	0.123	0.013	0.013		♂
IMPDH2		H0Y4R1																0.125	0.008	0.008	0.123	0.026	0.026	0.228		
IQGAP1		P46940																0.377	0.059	0.125	0.219	0.219	0.051	0.441		
ITGB4	Beta-4D	P16144-4																0.059	0.005	0.005	0.026	0.026	0.013	0.228		
ITIH2		Q5T985																0.174	0.059	0.125	0.349	0.349	0.026	0.148		♂
ITIH4		B2RMS9																0.038	0.005	0.024	0.123	0.026	0.026	0.051		
IVL		P07476																0.174	0.005	0.005	0.349	0.026	0.013	0.148		
KARS1		Q15046																0.301	0.024	0.024	0.349	0.219	0.013	0.327		♂
KHDRBS1		Q07666																0.458	0.005	0.008	0.123	0.026	0.013	0.148		
KHSRP		Q92945																0.125	0.005	0.024	0.500	0.026	0.013	0.051		
KIF5B-ALK	fusion	C1PHA2																0.377	0.005	0.005	0.349	0.026	0.013	0.228		
KNG1		B4E1C2																0.005	0.005	0.087	0.026	0.026	0.013	0.013		
KPNB1		Q14974																0.087	0.125	0.008	0.219	0.026	0.441	0.090	♀	
KRT1		H6VRG2																0.232	0.014	0.038	0.061	0.026	0.051	0.441	♀	
KRT10		A0A1B0GVI																0.059	0.005	0.024	0.026	0.026	0.013	0.228		
KRT10		P13645																0.174	0.008	0.024	0.061	0.026	0.026	0.441		
KRT14		P02533																0.174	0.232	0.038	0.349	0.349	0.228	0.051		

Gene	Isoform	Uniprot ID	volunteers					patients																		
								uninvolved					lesional													
			females			males		females		males			females		males											
			FH1	FH2	FH3	FH4	FH5	FU1	FU2	MU1	MU2	MU3	FL1	FL2	ML1	ML2	ML3	uninvolved vs. healthy skin	lesional vs. healthy skin	uninvolved vs. lesional skin	uninvolved female vs. healthy skin	lesional female vs healthy skin	uninvolved male vs. healthy skin	lesional male vs. healthy skin	DEPs, ♀ only	DEPs, ♂ only
KRT16		P08779																0.174	0.008	0.005	0.219	0.026	0.026	0.228		
KRT17		Q04695																0.301	0.059	0.014	0.219	0.061	0.148	0.441		
KRT19		P08727																0.458	0.059	0.014	0.219	0.026	0.051	0.090	♀	
KRT23		A0A024R1X																0.087	0.005	0.005	0.123	0.026	0.013	0.148		
KRT5		P13647																0.232	0.059	0.059	0.219	0.061	0.148	0.327		
KRT6A		P02538																0.301	0.014	0.008	0.349	0.026	0.051	0.327	♀	
KRT72		H0YHD9																0.125	0.059	0.125	0.219	0.026	0.228	0.148	♀	
KRT77		Q0IIN1																0.059	0.008	0.008	0.123	0.026	0.026	0.090		
KRT80	2	Q6KB66-2																0.458	0.008	0.014	0.123	0.026	0.026	0.228		
KRTDAP		P60985																0.059	0.024	0.174	0.349	0.026	0.090	0.026		
KTN1	2	Q86UP2-2																0.301	0.038	0.005	0.349	0.123	0.051	0.327		
LAD1		O00515																0.301	0.024	0.005	0.219	0.123	0.026	0.090		♂
LAP3	2	P28838-2																0.005	0.005	0.005	0.026	0.026	0.013	0.013		
LARS1	2	Q9P2J5-2																0.024	0.059	0.008	0.219	0.123	0.090	0.013		♂
LASP1		A0A024R1S																0.377	0.087	0.024	0.500	0.349	0.051	0.327		
LCN2	2	P80188-2																0.174	0.008	0.005	0.349	0.026	0.026	0.148		
LDHA		P00338																0.377	0.005	0.005	0.061	0.026	0.228	0.441	♀	
LDHB		Q5U077																0.458	0.014	0.008	0.349	0.123	0.013	0.327		♂
LGALS3		Q86TY5																0.232	0.024	0.008	0.219	0.061	0.051	0.327		
LGALS7		P47929																0.174	0.087	0.174	0.026	0.061	0.228	0.441	♀	
liposarcon	fusion	B4DHN0																0.301	0.059	0.014	0.349	0.123	0.228	0.327		
LMNA		P02545																0.008	0.005	0.024	0.026	0.026	0.013	0.026		
LMNB1		P20700																0.458	0.038	0.005	0.219	0.219	0.026	0.327		♂
LMNB2		Q03252																0.038	0.014	0.232	0.123	0.026	0.051	0.051	♀	
LRG1		P02750																0.008	0.005	0.087	0.061	0.026	0.013	0.013		
LRPPRC		E5KNY5																0.232	0.059	0.014	0.123	0.123	0.090	0.441		
LRRC59		Q96AG4																0.174	0.005	0.005	0.026	0.026	0.013	0.441		
LTA4H		A0A140VK2																0.174	0.024	0.024	0.219	0.219	0.013	0.228		♂
LTF		A0A161I202																0.174	0.087	0.005	0.123	0.061	0.228	0.327		
LUM		Q53FV4																0.232	0.005	0.005	0.349	0.026	0.013	0.228		
LY6D		Q14210																0.377	0.005	0.005	0.061	0.026	0.013	0.228		
MAGOHB		F5H6N1																0.377	0.008	0.059	0.061	0.026	0.026	0.051		
MAP4		E7EVA0																0.174	0.301	0.024	0.500	0.500	0.228	0.090		
MAPK1		B4DHN0																0.377	0.174	0.125	0.349	0.026	0.228	0.327	♀	
MAT		A0A140VJP																0.458	0.008	0.024	0.349	0.026	0.026	0.441		
MATR3		A8MXP9																0.059	0.024	0.125	0.123	0.123	0.026	0.090		♂
MDH1	2	P40925-2																0.301	0.174	0.008	0.349	0.219	0.228	0.148		
MDH2		Q75MT9																0.174	0.038	0.014	0.219	0.123	0.051	0.228		
ME1		P48163																0.458	0.008	0.024	0.500	0.061	0.013	0.441		♂
MRCL3		P19105																0.301	0.087	0.038	0.500	0.026	0.327	0.228	♀	
MSN		P26038																0.301	0.232	0.005	0.349	0.219	0.327	0.327		
MTHFD1		V9GYY3																0.174	0.005	0.014	0.026	0.026	0.013	0.441		
MTPN		C9JL85																0.174	0.125	0.014	0.219	0.349	0.090	0.228		
MTPN		P58546																0.125	0.125	0.087	0.026	0.026	0.441	0.441	♀	
MVP		Q14764																0.458	0.038	0.014	0.219	0.061	0.090	0.228		
MX1		P20591																0.458	0.008	0.005	0.349	0.061	0.013	0.441		♂
MYH11		A0A024QZJ																0.301	0.377	0.087	0.123	0.349	0.228	0.441		
MYH14		Q7Z406																0.005	0.005	0.174	0.026	0.026	0.013	0.013		
MYH9		A0A024R1N																0.059	0.174	0.005	0.123	0.349	0.148	0.090		
MYL1		Q53R15																0.232	0.125	0.301	0.500	0.026	0.441	0.148	♀	
MYO1D		J3QRN6																0.024	0.008	0.005	0.061	0.026	0.026	0.051		
Myosin-reactive immuno		Q9UL85																0.087	0.038	0.024	0.219	0.061	0.090	0.090		
Myosin-reactive immuno		Q9UL78																0.174	0.038	0.024	0.500	0.123	0.051	0.090		
NAA10	2	P41227-2																0.232	0.087	0.038	0.123	0.061	0.228	0.441		
NACA		F8VZJ2																0.059	0.005	0.005	0.500	0.026	0.013	0.013		
NAGK		Q9UJ70																0.174	0.014	0.005	0.349	0.061	0.026	0.148		♂
NANS		Q9NR45																0.059	0.059	0.024	0.219	0.026	0.228	0.051	♀	
NAP1L4		A0A2U9QGI																0.232	0.005	0.038	0.123	0.026	0.013	0.441		
NAPA		P54920																0.377	0.005	0.005	0.123	0.026	0.013	0.090		
NARS1		O43776																0.125	0.005	0.005	0.219	0.026	0.013	0.013		
NCCRP1		Q6ZVX7																0.125	0.005	0.005	0.349	0.026	0.013	0.090		
NCL		P19338																0.377	0.005	0.005	0.219	0.026	0.013	0.441		
NDPK		A0A384MTV																0.024	0.059	0.005	0.123	0.061	0.148	0.026		♂
NDRG1		Q92597																0.232	0.125	0.014	0.500	0.123	0.228	0.148		
NHP2L1		Q6FHM6																0.301	0.014	0.125	0.349	0.123	0.013	0.148		♂
NME2	3	P22392-2																0.301	0.024	0.005	0.500	0.061	0.051	0.228		
NNMT		Q6FH49																0.125	0.024	0.174	0.349	0.026	0.090	0.090	♀	
NPEPPS		P55786																0.377	0.005	0.005	0.349	0.026	0.013	0.441		
NPM1		A0A0S2Z49																0.377	0.005	0.005	0.500	0.026	0.013	0.327		
NSF		I3L0N3																0.377	0.014	0.008	0.349	0.026	0.051	0.228	♀	
NSFL1C		Q53FF5																0.301	0.038	0.008	0.219	0.123	0.051	0.441		
NT5C3		A0A090N7U																0.377	0.005	0.005	0.219	0.026	0.013	0.148		
NUCB1		Q53GX6																0.024	0.005	0.024	0.026	0.026	0.013	0.090		
NUCKS1	2	Q9H1E3-2																0.301	0.059	0.005	0.349	0.123	0.090	0.327		
NUTF2		P61970																0.174	0.059	0.059	0.349	0.026	0.228	0.148	♀	
OGN		A8K0R3																0.232	0.005	0.059	0.349	0.026	0.013	0.228		
OLA1		Q9NTK5																0.174	0.059	0.125	0.123	0.026	0.228	0.013		
OLFML1		Q6UWY5																0.232	0.038	0.014	0.219	0.061	0.090	0.327		
OLFML3		M1LAK4																0.232	0.038	0.024	0.349	0.061	0.090	0.090		
ORM1		P02763																0.008	0.005	0.125	0.061	0.026	0.013	0.013		
OTUB1		F5GYN4																0.377	0.038	0.038	0.500	0.026	0.148	0.327	♀	
OXCT		Q6IAV5																0.174	0.232	0.008	0.123	0.500	0.148	0.327		
P4HB		P07237																0.059	0.005	0.005	0.349	0.026	0.013	0.026		
PA2G4		Q9UQ80																0.458	0.005	0.005	0.219	0.026	0.013	0.327		
PABPC1		P11940																0.301	0.008	0.005	0.349	0.026	0.026	0.148		
PAFAH1B1		P43034																0.301	0.005	0.005	0.349	0.026	0.013	0.148		
PAICS		P22234																0.125	0.005	0.005	0.061	0.026	0.013	0.327		
PARK7		Q99497																0.301	0.087	0.125	0.219	0.500	0.026	0.441		♂
PARP1		P09874																0.458	0.087	0.087	0.219	0.500	0.026	0.228		♂
PBEF1		A0A024R71																0.125	0.005	0.005	0.123	0.026	0.013	0.228		
PC4		Q6IBA2																0.232	0.024	0.005	0.219	0.061	0.051	0.327		
PCBP1		Q53SS8																0.087	0.024	0.008	0.219	0.061	0.051	0.090		
PCBP2	4	Q15366-4																0.008	0.005	0.005	0.061	0.026	0.013	0.013		
PCNA		Q6FI35																0.024	0.038	0.005	0.026	0.026	0.148	0.090	♀	
PCYOX1		Q9UHG3																0.232	0.232	0.377	0.026	0.219	0.051	0.327	♀	
PDHB	3	P11177-3																0.232	0.005	0.174	0.219	0.026	0.013	0.327		
PDIA2		B3KQT9																0.059	0.174	0.008	0.123	0.123	0.327	0.090		
PDIA6	3	Q15084-3																0.458	0.059	0.005	0.500	0.123	0.090	0.441		
PDLIM1		O00151																0.301	0.014	0.038	0.123	0.123	0.013	0.441		♂
PFN1		P07737																0.301	0.125	0.005	0.123	0.219	0.148	0.441		
PGAM1		Q6P6D7																0.377	0.014	0.014	0.061	0.026	0.051	0.228	♀	
PGD	2	P52209-2																0.125	0.038	0.005	0.349	0.123	0.051	0.090		
PGK1		P00558																0.174	0.008	0.014	0.123	0.061	0.013	0.327		♂
PGM2		Q96G03																0.232	0.014	0.014	0.219	0.123	0.013	0.327		♂
PGRMC2		O15173																0.005	0.024	0.014	0.026	0.026	0.090	0.013		
PHB2		F5GY37																0.377	0.014	0.008	0.349	0.123	0.013	0.441		♂
PHGDH		O43175																0.059	0.024	0.005	0.123	0.026	0.090	0.090	♀	
PIG59		A8YXX4																0.232	0.005	0.008	0.500	0.026	0.013	0.148		
PITPNA		Q00169																0.038	0.014	0.005	0.026	0.026	0.051	0.148	♀	
PITPNB		P48739																0.458	0.005	0.005	0.349	0.026	0.013	0.441		
PKM		P14618																0.301	0.038	0.005	0.123	0.123	0.051	0.441		
PKP1		A0A024R95																0.377	0.174	0.232	0.123	0.026	0.441	0.090	♀	
PLA2G4D		Q86XP0																0.301	0.005	0.005	0.500	0.026	0.013	0.228		
PLG		B2R7F8																0.005	0.059	0.301	0.026	0.219	0.051	0.013		
PLS3		Q53GY0																0.232	0.059	0.024	0.123	0.219	0.051	0.441		
PML		P29590																0.087	0.087	0.059	0.123	0.349	0.013	0.148		♂
PNP		P00491																0.005	0.005	0.005	0.026	0.026	0.013	0.013		
POF1B	1	Q8WVV4-1																0.087	0.458	0.377	0.349	0.123	0.148	0.013		♂
POR		P16435																0.377	0.038	0.038	0.349	0.026	0.148	0.228	♀	
POSTN	5	Q15063-5																0.038	0.008	0.087	0.026	0.026	0.026	0.148		
PPA1		Q15181																0.232	0.024	0.005	0.061	0.123	0.026	0.441		♂
PPIB		P23284																0.038	0.038	0.005	0.349	0.061	0.090	0.013		♂
PPL		K7EKI8																0.301	0.125	0.125	0.349	0.500	0.051	0.148		
PPP1CA		P62136																0.458	0.014	0.024	0.500	0.123	0.013	0.441		♂
PPP2R1A		A8K7B7																0.232	0.005	0.005	0.219	0.026	0.013	0.327		
PRDX1		Q06830																0.458	0.005	0.008	0.349	0.026	0.013	0.327		
PRDX2		P32119																0.014	0.232	0.038	0.026	0.219	0.327	0.051	♀	
PRDX3	2	P30048-2																0.232	0.301	0.038	0.349	0.219	0.441	0.090		
PRDX4		Q13162																0.059	0.059	0.005	0.123	0.123	0.090	0.090		
PRELP		Q6FHG6																0.377	0.014	0.014	0.349	0.061	0.026	0.228		♂
PRKACA		P17612																0.232	0.232	0.024	0.219	0.349	0.228	0.327		
PRKDC		P78527																0.024	0.005	0.005	0.219	0.026	0.013	0.013		
PRPS1		B1ALA9																0.024	0.038	0.301	0.026	0.349	0.013	0.090		
PS1TP5BP1		Q1KLZ0																0.087	0.059	0.174	0.349	0.026	0.228	0.051	♀	
PSAT1		A0A024R28																0.301	0.174	0.008	0.349	0.123	0.327	0.327		
PSMA1		P25786																0.377	0.038	0.005	0.219	0.123	0.051	0.441		
PSMA3	2	P25788-2																0.458	0.005	0.005	0.349	0.026	0.013	0.441		
PSMA4		P25789																0.059	0.014	0.005	0.219	0.061	0.026	0.051		♂
PSMA6		PSMA6																0.125	0.005	0.005	0.123	0.026	0.013	0.228		
PSMA7		O14818																0.059	0.038	0.014	0.123	0.123	0.051	0.090		
PSMB2		P49721																0.059	0.087	0.125	0.123	0.500	0.026	0.090		♂
PSMB3		A0A087WU																0.125	0.038	0.087	0.123	0.349	0.013	0.228		♂
PSMB4		P28070																0.087	0.125	0.024	0.123	0.123	0.228	0.148		
PSMB5		P28074																0.059	0.059	0.059	0.500	0.026	0.228	0.013		
PSMB6		Q6IAT9																0.377	0.174	0.038	0.219	0.061	0.441	0.441		
PSMB7		Q86U62																0.024	0.005	0.005	0.026	0.026	0.013	0.090		
PSMB8		Q6FHU0																0.059	0.005	0.008	0.500	0.026	0.013	0.013		
PSMC1	2	P62191-2																0.125	0.024	0.059	0.219	0.026	0.090	0.148	♀	
PSMC2		P35998																0.377	0.008	0.008	0.500	0.026	0.026	0.327		
PSMC3		R4GNH3																0.458	0.008	0.014	0.500	0.026	0.026	0.441		
PSMC4		P43686																0.174	0.005	0.005	0.349	0.026	0.013	0.148		
PSMC5	2	P62195-2																0.038	0.014	0.005	0.026	0.123	0.013	0.148		
PSMD11		O00231																0.087	0.005	0.005	0.349	0.026	0.013	0.051		
PSMD12	2	O00232-2																0.458	0.038	0.024	0.349	0.219	0.026	0.441		♂
PSMD13		B4DJ66																0.087	0.125	0.014	0.026	0.061	0.327	0.327	♀	
PSMD2		Q13200																0.087	0.005	0.005	0.123	0.026	0.013	0.148		
PSMD3		O43242																0.301	0.038	0.038	0.500	0.026	0.148	0.228	♀	
PSMD6		Q15008																0.458	0.059	0.024	0.123	0.026	0.228	0.148	♀	
PSMD7		P51665																0.014	0.059	0.005	0.026	0.061	0.148	0.051	♀	
PSME1	3	Q06323-3																0.024	0.005	0.005	0.061	0.026	0.013	0.051		
PSME2		H0YM70																0.458	0.005	0.005	0.349	0.026	0.013	0.441		
PTBP1		P26599																0.174	0.005	0.005	0.500	0.026	0.013	0.090		
PTMA		B8ZZQ6																0.377	0.125	0.024	0.123	0.219	0.148	0.327		
PYGB		P11216																0.059	0.125	0.377	0.026	0.500	0.051	0.228	♀	
PYGL	2	P06737-2																0.125	0.024	0.008	0.349	0.026	0.090	0.090	♀	
QARS1		P47897																0.087	0.005	0.005	0.349	0.026	0.013	0.051		
RAB11B	2	Q15907-2																0.024	0.005	0.005	0.123	0.026	0.013	0.026		
RAB14		P61106																0.059	0.005	0.005	0.123	0.026	0.013	0.090		
RAB1A		P62820																0.008	0.005	0.005	0.026	0.026	0.013	0.026		
RAB1B		Q9H0U4																0.377	0.087	0.014	0.349	0.123	0.148	0.441		
RAB2		A0A024R7V																0.059	0.008	0.008	0.026	0.026	0.026	0.228		
RAB5C		A0A024R1U																0.087	0.005	0.005	0.123	0.026	0.013	0.148		
RAB6A		P20340																0.301	0.014	0.014	0.061	0.061	0.026	0.327		♂
RAB7A		A0A158RFU																0.458	0.005	0.005	0.500	0.026	0.013	0.441		
RAC1		A4D2P1																0.087	0.024	0.008	0.123	0.123	0.026	0.148		♂
RACK1		P63244																0.458	0.005	0.005	0.349	0.026	0.013	0.441		
RAD23B		P54727																0.125	0.125	0.232	0.219	0.349	0.026	0.148		♂
RALY		Q5QPL9																0.024	0.125	0.038	0.219	0.219	0.148	0.013		♂
RAN		P62826																0.458	0.038	0.024	0.123	0.061	0.090	0.228		
RARS1		P54136																0.174	0.038	0.038	0.500	0.349	0.013	0.090		♂
RBBP4	3	Q09028-3																0.059	0.014	0.005	0.123	0.123	0.013	0.090		♂
RBM3		A0A024QYX																0.174	0.059	0.059	0.123	0.500	0.013	0.327		♂
RBMX		P38159																0.232	0.087	0.014	0.061	0.349	0.051	0.441		
RECQL		A0A024RAV																0.232	0.024	0.008	0.500	0.061	0.051	0.148		
REEP1		B7Z6B3																0.377	0.059	0.008	0.219	0.123	0.090	0.441		
RNH1		P13489																0.038	0.232	0.005	0.123	0.219	0.327	0.051		
RNPEP		A6NKB8																0.059	0.059	0.024	0.026	0.349	0.026	0.228		
RPL10		X5D2T3																0.301	0.024	0.014	0.500	0.026	0.090	0.228	♀	
RPL10A		P62906																0.125	0.005	0.005	0.123	0.026	0.013	0.228		
RPL11		Q5VVD0																0.301	0.038	0.038	0.219	0.026	0.148	0.441	♀	
RPL13		Q6NZ55																0.301	0.024	0.024	0.123	0.219	0.013	0.441		♂
RPL13A		P40429																0.301	0.014	0.005	0.061	0.026	0.051	0.327	♀	
RPL14		Q6IPH7																0.301	0.014	0.008	0.123	0.026	0.051	0.441	♀	
RPL15		A0A024R2Q																0.458	0.005	0.005	0.500	0.026	0.013	0.441		
RPL17		J3QQT2																0.377	0.008	0.005	0.349	0.061	0.013	0.228		♂
RPL18		A0A024QZD																0.301	0.005	0.005	0.123	0.026	0.013	0.441		
RPL18A		M0R117																0.087	0.005	0.005	0.123	0.026	0.013	0.148		
RPL19		J3QR09																0.125	0.038	0.059	0.219	0.349	0.013	0.148		♂
RPL21		Q6IAX2																0.232	0.024	0.087	0.500	0.061	0.051	0.148		
RPL22		K7ERI7																0.458	0.059	0.014	0.349	0.061	0.148	0.441		
RPL23		P62829																0.377	0.038	0.038	0.219	0.349	0.013	0.441		♂
RPL23A		P62750																0.174	0.024	0.005	0.123	0.026	0.090	0.327	♀	

			volunteers					patients																
								uninvolved					lesional											
			females			males		females		males			females		males									
Gene	Isoform	Uniprot ID	FH1	FH2	FH3	FH4	FH5	FU1	FU2	MU1	MU2	MU3	FL1	FL2	ML1	ML2	ML3	uninvolved vs. healthy skin	lesional vs. healthy skin	uninvolved vs. lesional skin	uninvolved female vs. healthy skin	lesional female vs healthy skin	uninvolved male vs. healthy skin	lesional male vs. healthy skin	DEPs, ♀ only	DEPs, ♂ only
RPL26		Q6IBH6																0.301	0.005	0.005	0.349	0.026	0.013	0.327		
RPL27		A0A024R1V																0.125	0.005	0.005	0.219	0.026	0.013	0.148		
RPL27A		P46776																0.301	0.005	0.005	0.349	0.026	0.013	0.148		
RPL28		P46779																0.174	0.024	0.014	0.123	0.026	0.090	0.327	♀	
RPL29		A0A024R32																0.377	0.008	0.005	0.500	0.026	0.026	0.327		
RPL3		P39023																0.301	0.005	0.005	0.123	0.026	0.013	0.441		
RPL30		E5RI99																0.174	0.014	0.038	0.349	0.026	0.051	0.148	♀	
RPL32		D3YTB1																0.377	0.008	0.008	0.123	0.061	0.013	0.327		♂
RPL35		P42766																0.377	0.005	0.005	0.500	0.026	0.013	0.327		
RPL35A		P18077																0.301	0.014	0.005	0.349	0.061	0.026	0.148		♂
RPL36		Q9Y3U8																0.059	0.005	0.014	0.123	0.026	0.013	0.090		
RPL37A		P61513																0.087	0.014	0.038	0.219	0.123	0.013	0.090		♂
RPL38		A0A024R8P																0.301	0.458	0.008	0.349	0.349	0.327	0.327		
RPL39		P62891																0.005	0.005	0.008	0.026	0.026	0.013	0.013		
RPL4		P36578																0.377	0.005	0.005	0.219	0.026	0.013	0.441		
RPL5		A2RUM7																0.038	0.008	0.005	0.061	0.026	0.026	0.090		
RPL6		Q8TBK5																0.087	0.005	0.005	0.061	0.026	0.013	0.228		
RPL7		P18124																0.087	0.005	0.008	0.500	0.026	0.013	0.026		
RPL7A		P62424																0.301	0.024	0.014	0.349	0.061	0.051	0.327		
RPL8		P62917																0.458	0.008	0.005	0.349	0.061	0.013	0.327		♂
RPL9		Q53Z07																0.232	0.005	0.005	0.500	0.026	0.013	0.148		
RPLP0		P05388																0.087	0.005	0.005	0.349	0.026	0.013	0.051		
RPLP2		A0A024RCA																0.301	0.005	0.005	0.219	0.026	0.013	0.441		
RPN1		Q6IBR0																0.458	0.024	0.008	0.219	0.061	0.051	0.228		
RPN2	2	P04844-2																0.377	0.005	0.005	0.349	0.026	0.013	0.228		
RPS11		P62280																0.125	0.005	0.005	0.219	0.026	0.013	0.148		
RPS12		P25398																0.125	0.232	0.125	0.026	0.500	0.148	0.441	♀	
RPS13		P62277																0.377	0.005	0.005	0.219	0.026	0.013	0.441		
RPS14		A0A2R8Y81																0.232	0.005	0.005	0.123	0.026	0.013	0.441		
RPS15		K7EM56																0.301	0.014	0.024	0.349	0.026	0.051	0.148	♀	
RPS15A		P62244																0.458	0.005	0.005	0.349	0.026	0.013	0.441		
RPS16		P62249																0.301	0.014	0.005	0.349	0.061	0.026	0.148		♂
RPS17		P08708																0.377	0.005	0.005	0.349	0.026	0.013	0.228		
RPS18		P62269																0.458	0.005	0.005	0.500	0.026	0.013	0.441		
RPS19		B0ZBD0																0.458	0.014	0.014	0.349	0.026	0.051	0.327	♀	
RPS2		P15880																0.458	0.005	0.005	0.500	0.026	0.013	0.441		
RPS20		P60866																0.059	0.005	0.005	0.123	0.026	0.013	0.090		
RPS21		Q8WVC2																0.059	0.005	0.005	0.061	0.026	0.013	0.148		
RPS23		A8K517																0.174	0.005	0.005	0.123	0.026	0.013	0.327		
RPS24		P62847-2																0.458	0.005	0.005	0.349	0.026	0.013	0.441		
RPS25		P62851																0.301	0.005	0.005	0.349	0.026	0.013	0.327		
RPS27		P42677																0.301	0.014	0.005	0.123	0.026	0.051	0.441	♀	
RPS28		B2R4R9																0.059	0.014	0.005	0.123	0.061	0.026	0.090		♂
RPS3		P23396																0.087	0.005	0.005	0.123	0.026	0.013	0.148		
RPS3A		Q6NXR8																0.087	0.005	0.005	0.026	0.026	0.013	0.327		
RPS4X		Q96IR1																0.232	0.008	0.005	0.123	0.026	0.026	0.441		
RPS5		M0R0F0																0.087	0.005	0.005	0.219	0.026	0.013	0.090		
RPS6		Q96DV6																0.125	0.005	0.005	0.349	0.026	0.013	0.090		
RPS7		P62081																0.377	0.014	0.005	0.500	0.026	0.051	0.327	♀	
RPS8		Q5JR94																0.301	0.014	0.008	0.219	0.026	0.051	0.441	♀	
RPS9		A0A024R4M																0.377	0.008	0.005	0.349	0.026	0.026	0.228		
RPSA		C9J9K3																0.458	0.005	0.008	0.349	0.026	0.013	0.327		
RRBP1		A1A5C4																0.059	0.087	0.005	0.061	0.123	0.148	0.148		
RTCB		Q9Y3I0																0.301	0.024	0.005	0.123	0.061	0.051	0.441		
RUVBL1		B5BUB1																0.377	0.038	0.014	0.349	0.219	0.026	0.441		♂
RUVBL2		Q9Y230																0.301	0.038	0.038	0.123	0.349	0.013	0.441		♂
S100A10		P60903																0.059	0.024	0.377	0.123	0.026	0.090	0.090	♀	
S100A12		P80511																0.038	0.232	0.014	0.123	0.123	0.441	0.051		
S100A16		Q96FQ6																0.377	0.005	0.005	0.219	0.026	0.013	0.148		
S100A2		P29034																0.174	0.005	0.005	0.500	0.026	0.013	0.090		
S100A4		P26447																0.008	0.174	0.458	0.026	0.349	0.148	0.026		
S100A6		R4GN98																0.059	0.005	0.008	0.219	0.026	0.013	0.051		
S100A7		P31151																0.008	0.005	0.005	0.026	0.026	0.013	0.026		
S100A7A		Q86SG5																0.087	0.005	0.008	0.500	0.026	0.013	0.026		
S100A8		P05109																0.059	0.005	0.005	0.123	0.026	0.013	0.090		
S100A9		P06702																0.125	0.005	0.005	0.219	0.026	0.013	0.148		
S100P		P25815																0.232	0.008	0.005	0.219	0.026	0.026	0.327		
SAMHD1		Q9Y3Z3																0.059	0.024	0.014	0.123	0.123	0.026	0.090		♂
SARS		Q0VGA5																0.458	0.038	0.024	0.349	0.026	0.148	0.441	♀	
SBSN		Q6UWP8																0.301	0.005	0.014	0.349	0.026	0.013	0.148		
SBSN	2	Q6UWP8-2																0.301	0.005	0.005	0.123	0.026	0.013	0.051		
SCCA1/SC	fusion	Q5K684																0.087	0.005	0.005	0.123	0.026	0.013	0.148		
SCCA2/SC	fusion	Q5K634																0.005	0.005	0.005	0.026	0.026	0.013	0.013		
SEC13		P55735																0.232	0.087	0.014	0.123	0.349	0.051	0.441		
SEC22B		O75396																0.059	0.301	0.038	0.123	0.349	0.148	0.090		
SEC31A		D6REX3																0.005	0.024	0.005	0.026	0.123	0.026	0.013		
SELENBP1		Q13228																0.038	0.301	0.024	0.061	0.500	0.228	0.090		
SEPTIN11		D6RGI3																0.008	0.005	0.174	0.026	0.026	0.013	0.026		
SEPTIN7		Q16181																0.014	0.008	0.174	0.026	0.026	0.026	0.051		
SERBP1	3	Q8NC51-3																0.377	0.005	0.005	0.349	0.026	0.013	0.228		
SERPINA1		P01009																0.174	0.038	0.232	0.219	0.026	0.148	0.228	♀	
SERPINA3		A0A024R6P																0.024	0.005	0.458	0.219	0.026	0.013	0.013		
SERPINB1	2	Q96P63-2																0.059	0.174	0.014	0.061	0.500	0.090	0.148		
SERPINB3		P29508																0.038	0.005	0.005	0.219	0.026	0.013	0.026		
SERPINB5		A0A024R2B																0.174	0.005	0.014	0.349	0.026	0.013	0.148		
SERPINB6		A0A2R8YD1																0.014	0.174	0.458	0.061	0.026	0.441	0.026		
SERPINB7	2	O75635-2																0.377	0.008	0.014	0.500	0.026	0.026	0.327		
SERPINC1		A0A024R94																0.005	0.005	0.087	0.026	0.026	0.013	0.013		
SERPIND1		P05546																0.038	0.014	0.174	0.123	0.061	0.026	0.051		♂
SERPINF1		P36955																0.087	0.014	0.024	0.219	0.061	0.026	0.090		♂
SERPING1		A0A348GSH																0.059	0.024	0.174	0.123	0.123	0.026	0.090		♂
SERPINH1		P50454																0.008	0.038	0.059	0.061	0.123	0.051	0.013		♂
SF3A1		A0A024R1K																0.301	0.014	0.005	0.349	0.123	0.013	0.327		♂
SFN		P31947																0.377	0.024	0.059	0.123	0.061	0.051	0.090		
SFPQ		Q9BSV4																0.377	0.005	0.005	0.349	0.026	0.013	0.228		
SHMT2		Q5BJF5																0.087	0.024	0.024	0.026	0.219	0.013	0.327		
SKP1		E5RJR5																0.125	0.008	0.024	0.500	0.026	0.026	0.051		
SLC25A11		I3L1P8																0.125	0.005	0.014	0.219	0.026	0.013	0.148		
SLC25A3		Q00325																0.377	0.005	0.005	0.500	0.026	0.013	0.327		
SLC25A5		P05141																0.059	0.005	0.005	0.219	0.026	0.013	0.051		
SLC25A6		Q6I9V5																0.125	0.014	0.024	0.349	0.123	0.013	0.090		♂
SLC4A1		G4V2I9																0.005	0.059	0.024	0.026	0.123	0.090	0.013		
SNCA		Q4W5L2																0.005	0.087	0.008	0.026	0.349	0.051	0.013		
SNRNP70	2	P08621-2																0.087	0.174	0.008	0.026	0.349	0.148	0.327	♀	
SNRPD1		J3QLI9																0.059	0.014	0.038	0.026	0.123	0.013	0.228		
SNRPD2		P62316																0.087	0.008	0.014	0.123	0.061	0.013	0.148		♂
SNRPD3		P62318																0.458	0.024	0.024	0.349	0.061	0.051	0.441		
SNX2		O60749																0.038	0.301	0.087	0.123	0.123	0.441	0.051		
SOD		A0A140VJU																0.301	0.038	0.008	0.219	0.061	0.090	0.441		
SOD1		P00441																0.125	0.301	0.087	0.349	0.061	0.327	0.026		♂
SOD2		P04179																0.087	0.008	0.008	0.219	0.061	0.013	0.090		♂
SPCS2		E9PRB9																0.125	0.059	0.087	0.061	0.026	0.228	0.327	♀	
SPTA1		P02549																0.014	0.087	0.038	0.061	0.123	0.148	0.026		♂
SPTAN1		Q13813																0.005	0.005	0.059	0.026	0.026	0.013	0.013		
SPTB		P11277																0.024	0.174	0.059	0.061	0.123	0.327	0.051		
SPTBN1		B2ZZ89																0.005	0.005	0.014	0.026	0.026	0.013	0.013		
SPTBN2		O15020																0.301	0.087	0.125	0.061	0.026	0.327	0.327	♀	
SQOR		Q9Y6N5																0.174	0.005	0.005	0.349	0.026	0.013	0.148		
SRP14		P37108																0.377	0.038	0.059	0.349	0.219	0.026	0.228		♂
SRP9		P49458																0.174	0.174	0.038	0.349	0.349	0.051	0.148		
SRSF1		Q07955																0.087	0.005	0.005	0.219	0.026	0.013	0.090		
SRSF2		J3KP15																0.059	0.014	0.024	0.349	0.123	0.013	0.026		♂
SRSF3	2	P84103-2																0.174	0.014	0.014	0.219	0.026	0.051	0.228	♀	
SSR4		P51571																0.125	0.005	0.005	0.349	0.026	0.013	0.090		
STAT1		P42224																0.014	0.005	0.005	0.123	0.026	0.013	0.013		
STAT3	3	P40763-3																0.301	0.005	0.008	0.500	0.026	0.013	0.228		
STIP1		P31948																0.377	0.005	0.005	0.123	0.026	0.013	0.327		
STMN1		B5BU83																0.377	0.008	0.014	0.349	0.026	0.026	0.228		
STOM		P27105																0.014	0.125	0.174	0.026	0.349	0.090	0.051	♀	
SUCLG2		Q96I99																0.174	0.038	0.174	0.061	0.219	0.026	0.441		♂
SUI1		Q6IAV3																0.458	0.005	0.008	0.500	0.026	0.013	0.441		
SULT2B1	2	O00204-2																0.174	0.008	0.008	0.500	0.061	0.013	0.090		♂
SUMO2	2	P61956-2																0.301	0.014	0.008	0.500	0.123	0.013	0.228		♂
SYNCRIP		Q05CK9																0.232	0.005	0.005	0.219	0.026	0.013	0.327		
TACSTD2		P09758																0.232	0.087	0.087	0.061	0.026	0.327	0.441	♀	
TAGLN2		A0A384MTL																0.458	0.024	0.024	0.219	0.026	0.090	0.327	♀	
TALDO1		A0A140VK5																0.087	0.232	0.024	0.349	0.123	0.441	0.051		
TARDBP		Q13148																0.377	0.008	0.008	0.219	0.026	0.026	0.148		
TCP1		P17987																0.087	0.005	0.005	0.123	0.026	0.013	0.148		
TDRD11		B2R5U1																0.038	0.014	0.005	0.061	0.026	0.051	0.090	♀	
TGFBI		A0A0S2Z4Q																0.008	0.005	0.087	0.026	0.026	0.013	0.026		
TGM3		Q08188																0.005	0.005	0.005	0.026	0.026	0.013	0.013		
THBS1		A0A024R9Q																0.008	0.087	0.059	0.061	0.123	0.148	0.013		♂
THRAP3		A0A3B3ITZ9																0.301	0.024	0.024	0.123	0.026	0.090	0.441	♀	
TKT	2	P29401-2																0.232	0.125	0.005	0.349	0.219	0.148	0.228		
TMED10		A0A024R6I3																0.059	0.005	0.005	0.123	0.026	0.013	0.090		
TMEM43		A0A024R2F																0.174	0.125	0.377	0.061	0.026	0.441	0.441	♀	
TMSB10		P63313																0.087	0.059	0.005	0.219	0.061	0.148	0.090		
TMSB4X		A2VCK8																0.024	0.301	0.038	0.026	0.123	0.441	0.090	♀	
TNC		A7MBN2																0.458	0.038	0.005	0.349	0.123	0.051	0.327		
TNXB		A0A3B3ISX9																0.301	0.005	0.024	0.219	0.026	0.013	0.441		
TPI1		P60174																0.087	0.024	0.005	0.123	0.061	0.051	0.148		
TPM3		A0A0S2Z4G																0.014	0.024	0.005	0.026	0.061	0.051	0.051	♀	
TPM4		P67936																0.005	0.377	0.005	0.026	0.349	0.441	0.013		
TPP	2	O14773-2																0.087	0.024	0.377	0.500	0.123	0.026	0.026		♂
TPPP3		Q9BW30																0.174	0.008	0.008	0.349	0.026	0.026	0.148		
TPSAB1		Q6B051																0.125	0.038	0.087	0.219	0.061	0.090	0.148		
TPT1		P13693																0.377	0.008	0.005	0.500	0.026	0.026	0.327		
TRA1		Q5CAQ5																0.014	0.174	0.005	0.061	0.123	0.327	0.026		♂
TRA2B	3	P62995-3																0.301	0.024	0.059	0.349	0.219	0.013	0.148		♂
TREX2	2	Q9BQ50-2																0.014	0.005	0.005	0.061	0.026	0.013	0.026		
TRIM25		Q59GW5																0.458	0.005	0.005	0.500	0.026	0.013	0.441		
TRIM29		Q14134																0.059	0.232	0.024	0.123	0.349	0.228	0.090		
TTR		P02766																0.024	0.024	0.059	0.061	0.026	0.090	0.051	♀	
TUBA1B		P68363																0.458	0.059	0.024	0.500	0.349	0.026	0.441		♂
TUBA4A	4	P68366-2																0.174	0.005	0.038	0.500	0.026	0.013	0.090		
TUBB		Q5JP53																0.301	0.301	0.014	0.349	0.500	0.228	0.327		
TUBB2A		Q13885																0.301	0.174	0.038	0.219	0.061	0.441	0.441		
TUBB2C		Q8IZ29																0.377	0.014	0.008	0.500	0.026	0.051	0.327	♀	
TUFM		A0A384ME1																0.458	0.024	0.059	0.349	0.219	0.013	0.327		♂
TXNDC17		Q9BRA2																0.024	0.014	0.059	0.061	0.123	0.013	0.051		♂
TXNDC5		Q8NBS9																0.038	0.125	0.008	0.349	0.123	0.228	0.013		♂
TXNL1		O43396																0.038	0.087	0.008	0.026	0.026	0.327	0.148	♀	
TYMP		P19971																0.059	0.005	0.005	0.123	0.026	0.013	0.090		
UBA52		P62987																0.174	0.014	0.014	0.026	0.123	0.013	0.441		
UBE2N		P61088																0.458	0.038	0.005	0.349	0.061	0.090	0.441		
UCHL3		A0A087WTE																0.059	0.005	0.005	0.061	0.026	0.013	0.148		
UFM1		P61960																0.377	0.059	0.038	0.123	0.219	0.051	0.327		
UGDH	2	O60701-2																0.232	0.125	0.038	0.219	0.123	0.228	0.327		
UGP2		Q53QE9																0.301	0.038	0.024	0.349	0.123	0.051	0.327		
Uncharacterized		Q6MZU6																0.014	0.014	0.377	0.123	0.123	0.013	0.013		♂
Uncharacterized		Q6MZV7																0.024	0.038	0.458	0.123	0.349	0.013	0.026		♂
Uncharacterized		V9HW68																0.038	0.024	0.301	0.219	0.219	0.013	0.026		♂
Unidentified		Q53FT8																0.005	0.005	0.458	0.026	0.026	0.013	0.013		♂
Unidentified		Q53GF5																0.014	0.005	0.301	0.123	0.026	0.013	0.013		♂
Unidentified		B4E0S6																0.125	0.005	0.005	0.123	0.026	0.013	0.228		♂
Unidentified		Q5U0A0																0.232	0.014	0.038	0.349	0.123	0.013	0.228		
Unidentified		Q6PUJ7																0.377	0.005	0.005	0.123	0.026	0.013	0.327		
Unidentified		Q8TCD0																0.232	0.059	0.125	0.123	0.500	0.013	0.441		
Unidentified		A0A384P5V																0.301	0.059	0.059	0.219	0.500	0.013	0.441		♂
Unidentified		V9HWH1																0.458	0.024	0.014	0.500	0.219	0.013	0.441	♀	
Unidentified		V9HWJ7																0.087	0.014	0.005	0.123	0.061	0.026	0.148		♂
Unidentified		B4E1B2																0.301	0.014	0.005	0.219	0.026	0.051	0.441		
Unidentified		A0A384MD																0.024	0.059	0.038	0.061	0.123	0.090	0.051		
UQCRQ		O14949																0.232	0.059	0.038	0.123	0.123	0.090	0.441		
USP5	short	P45974-2																0.377	0.024	0.014	0.061	0.219	0.013	0.228		♂
VAMP8		Q9BV40																0.301	0.024	0.005	0.123	0.061	0.051	0.441		
VAPA		Q9P0L0																0.377	0.005	0.038	0.349	0.026	0.013	0.228		
VAT1		Q99536																0.125	0.059	0.174	0.219	0.026	0.228	0.148	♀	
VCAN	V3	P13611-4																0.059	0.038	0.174	0.061	0.061	0.090	0.148		
VCP		P55072																0.125	0.005	0.005	0.219	0.026	0.013	0.148		
VDAC1		P21796																0.301	0.014	0.005	0.349	0.061	0.026	0.327		♂
VDAC2		A0A0A0MR																0.125	0.038	0.174	0.219	0.123	0.051	0.148		

			volunteers					patients																		
								uninvolved					lesional													
			females			males		females		males			females		males											
Gene	Isoform	Uniprot ID	FH1	FH2	FH3	FH4	FH5	FU1	FU2	MU1	MU2	MU3	FL1	FL2	ML1	ML2	ML3	uninvolved vs. healthy skin	lesional vs. healthy skin	uninvolved vs. lesional skin	uninvolved female vs. healthy skin	lesional female vs healthy skin	uninvolved male vs. healthy skin	lesional male vs. healthy skin	DEPs, ♀ only	DEPs, ♂ only
VDAC3		Q9Y277																0.232	0.005	0.024	0.123	0.026	0.013	0.441		
VIM		P08670																0.014	0.005	0.014	0.123	0.026	0.013	0.013		
V-kappa-3		A0N5G5																0.232	0.024	0.038	0.349	0.061	0.051	0.090		
VPS4B		A0A024R2C																0.301	0.232	0.024	0.349	0.061	0.441	0.148		
WARS1		P23381																0.087	0.014	0.005	0.123	0.026	0.051	0.148	♀	
XRCC5		P13010																0.087	0.024	0.038	0.219	0.219	0.013	0.090		♂
XRCC6		A0A024R1N																0.377	0.005	0.014	0.349	0.026	0.013	0.441		
YARS		A0A0S2Z4R																0.174	0.005	0.005	0.026	0.026	0.013	0.441		
YBX1		P67809																0.125	0.005	0.005	0.349	0.026	0.013	0.090		
YWHAE		P62258																0.301	0.024	0.005	0.500	0.061	0.051	0.228		
YWHAG		P61981																0.174	0.014	0.005	0.500	0.026	0.051	0.090	♀	
YWHAH		A0A024R1K																0.059	0.024	0.125	0.061	0.123	0.026	0.148		♂
YWHAQ		P27348																0.174	0.024	0.005	0.219	0.061	0.051	0.228		
YWHAZ		D0PNI1																0.458	0.008	0.008	0.219	0.026	0.026	0.327		
																								**Total:**	123	177
